# Early neurodevelopment of HIV-exposed uninfected children in the era of antiretroviral therapy: a systematic review and meta-analysis

**DOI:** 10.1016/S2352-4642(22)00071-2

**Published:** 2022-06

**Authors:** Catherine J Wedderburn, Ella Weldon, Cesc Bertran-Cobo, Andrea M Rehman, Dan J Stein, Diana M Gibb, Shunmay Yeung, Andrew J Prendergast, Kirsten A Donald

**Affiliations:** a Department of Paediatrics and Child Health, Red Cross War Memorial Children's Hospital, University of Cape Town, Cape Town, South Africa; b The Neuroscience Institute, University of Cape Town, Cape Town, South Africa; c Department of Psychiatry and Mental Health, University of Cape Town, Cape Town, South Africa; d MRC Unit on Risk and Resilience in Mental Disorders, University of Cape Town, Cape Town, South Africa; e Department of Clinical Research, London School of Hygiene & Tropical Medicine, London, UK; f MRC International Statistics & Epidemiology Group, London School of Hygiene & Tropical Medicine, London, UK; g MRC Clinical Trials Unit, University College London, London, UK; h Blizard Institute, Queen Mary University of London, London, UK; i Zvitambo Institute for Maternal and Child Health Research, Harare, Zimbabwe

## Abstract

**Background:**

There are 15·4 million children who are HIV-exposed and uninfected worldwide. Early child development crucially influences later academic and socioeconomic factors. However, the neurodevelopmental outcomes of HIV-exposed uninfected (HEU) children in the era of maternal antiretroviral therapy (ART) remain unclear. We aimed to examine the effects of in-utero exposure to HIV and ART on child neurodevelopment.

**Methods:**

For this systematic review and meta-analysis, we searched MEDLINE, Embase, PubMed, Africa-Wide Information, PsycInfo, and Global Health databases from inception to May 27, 2020, for studies from the past two decades reporting neurodevelopment of HEU children aged 0–5 years compared with HIV-unexposed (HU) children (aim 1), and effects of different maternal ART regimens on neurodevelopment of HEU children (aim 2). We did narrative syntheses for both aims, and a random-effects meta-analysis of high-quality studies comparing HEU children and HU children, to obtain weighted pooled estimates of effect sizes. This study was registered with PROSPERO, CRD42018075910.

**Findings:**

We screened 35 527 records and included 45 articles from 31 studies. Overall, 12 (57%) of 21 studies comparing HEU children and HU children found worse neurodevelopment in HEU children in at least one domain. Study design and methodological quality were variable, with heterogeneity across populations. Meta-analysis included eight high-quality studies comparing 1856 HEU children with 3067 HU children at ages 12–24 months; among HEU children with available data, 1709 (99%) of 1732 were exposed to ART. HEU children had poorer expressive language (effect size –0·17 [95% CI –0·27 to –0·07], p=0·0013) and gross motor function (–0·13 [–0·20 to –0·07], p<0·0001) than HU children, but similar cognitive development (–0·06 [–0·19 to 0·06], p=0·34), receptive language development (–0·10 [–0·23 to 0·03], p=0·14), and fine motor skills (–0·05 [–0·15 to 0·06], p=0·36). Results suggested little or no evidence of an effect of specific maternal ART regimens on neurodevelopment; study heterogeneity prevented meta-analysis.

**Interpretation:**

HEU children are at risk of subtle impairments in expressive language and gross motor development by age 2 years. We found no consistent effect of maternal ART regimens analysed, although evidence was scarce. We highlight the need for large high-quality longitudinal studies to assess the neurodevelopmental trajectories of HEU children and to investigate underlying mechanisms to inform intervention strategies.

**Funding:**

Wellcome Trust and Medical Research Council.

## Introduction

Widespread access to antiretroviral therapy (ART) in pregnancy has substantially reduced vertical HIV transmission, meaning most children born to mothers with HIV are HIV-exposed and uninfected. There are an estimated 15·4 million HIV-exposed uninfected (HEU) children worldwide, comprising over 20% of annual births in some high HIV-burden countries. Disparities in early-life mortality and morbidity are evident between HEU children and HIV-unexposed (HU) children, and concerns have been raised regarding the effects of HIV and ART exposure on neurodevelopment.^1^, ^2^

Early child development forms the basis of future academic achievement and socioeconomic outcomes.^3^ The Sustainable Development Goals recognise the importance of child neurodevelopment, since the early years are foundational for brain development.^4^ It is important to understand the manifold risk factors for impaired development to inform intervention strategies that enhance child neurodevelopment potential. However, neurodevelopment is difficult to measure at young ages, making it challenging to interpret findings from single studies.

Existing literature suggests that HEU children might be at risk for adverse cognitive, language, and motor outcomes compared with HU children. However, previous systematic reviews describing neurodevelopmental delay include multiple reports from before widespread access to ART and few studies from countries with generalised HIV epidemics.^5^, ^6^, ^7^ Although two meta-analyses have been done, both were restricted to studies that used the Bayley Scales of Infant and Toddler Development, some with high risk of bias.^7^, ^8^ Further, in one meta-analysis, all studies from outside the USA were classified as low quality because of potential confounding and small sample sizes,^7^ thereby limiting understanding of outcomes of HEU children in regions of high HIV prevalence.

**Research in context**

**Evidence before this study**

Children who are HIV-exposed and uninfected represent a growing global population. The neurodevelopmental outcomes of this group of children in the era of antiretroviral therapy (ART) remain unclear; however, evidence is emerging from recent studies. Further information is needed to understand the nature of early neurodevelopment in this population to inform provision of care to improve outcomes. On searching the literature, we identified two meta-analyses but these were limited by the low number of included studies, including some with high risk of bias, thereby leaving a gap in our understanding of the neurodevelopmental outcomes of HIV-exposed uninfected (HEU) children in regions of high HIV prevalence. Individual studies were often limited by sample size and although previous reviews have been done, the majority have included studies from before widespread access to ART or have few studies from sub-Saharan Africa where the highest burden of HIV exists. We are unaware of any systematic reviews that have assessed the effect of different maternal ART regimens or examined imaging and head circumference alongside neurodevelopmental assessments. There is a need for an updated and focused synthesis of data for HIV and ART exposure on neurodevelopment in the ART era.

**Added value of this study**

Our systematic review included 45 articles (from 31 studies) building on previous reports by contributing updated data following widespread access to ART. Our meta-analysis combined larger, high quality studies, mostly from sub-Saharan Africa, reflective of the current population of HEU children. We found that HEU children have worse expressive language and gross motor development compared with HIV-unexposed children with small effect sizes, but with similar cognitive, receptive language, and fine motor skills. To our knowledge, this is the first systematic review to assess effects of different maternal ART regimens on the neurodevelopment of HEU children. We found that few studies explored the effects of ART, but the scarce evidence suggests that there is little, if any, effect of the specific ART regimens or drug classes assessed on neurodevelopmental outcomes, although concerns were raised for efavirenz and atazanavir.

**Implications of all the available evidence**

HEU children are at risk for subtle impairments in expressive language and gross motor development in early life. Although effect sizes were relatively small, the large number of HEU children worldwide means that even these subtle deficits might have a substantial effect in high-HIV burden countries, particularly in environments with multiple overlapping risk factors. Supporting these children to thrive might require interventions that focus on expressive language and gross motor skills in early childhood. Future research with large, high-quality, longitudinal studies is needed to examine outcomes at older ages and to investigate underlying mechanisms, with consistent methodology and standardised tools across settings to inform prevention and intervention strategies.

Access to triple-drug ART during pregnancy and breastfeeding has expanded, leading to improved maternal survival and higher breastfeeding rates, which might influence neurodevelopment.^9^ Several studies from after the rollout of ART have reported that HEU children remain at risk of delayed neurodevelopment;^10^, ^11^ however, other studies found no differences when compared with HU children.^12^, ^13^ Separately, in-utero exposure to ART has been associated with adverse neurodevelopment.^14^ Due to heterogeneity across studies and populations, including differences in maternal ART use and neurodevelopmental assessment tools, uncertainty remains regarding the outcomes of HEU children in the present day. Our first aim was to examine the effect of in-utero HIV exposure on child neurodevelopment through a comparison of HEU children and HU children, and our second aim was to investigate the effect of in-utero ART exposure on the neurodevelopment of HEU children.

## Methods

### Search strategy and selection criteria

We searched MEDLINE, Pubmed, Embase, PsychINFO, Global Health, and Africa-Wide Information without language restrictions from database inception to May 27, 2020. We used search terms for “child”, “neurodevelopment”, and “HIV/ART”, which were adapted for each database. MeSH headings were also used in MEDLINE, and Emtree terms in Embase, combined with database-specific filters. The search strategy and search terms are in the appendix (pp 3–6). The reference lists and citations of eligible papers were searched for additional studies.

We defined our inclusion and exclusion criteria in line with the Population, Exposure, Comparator, Outcomes^15^, ^16^ framework (appendix p 7). Eligible studies included HEU children aged 0–5 years born after Jan 1, 2000. We excluded studies in which antiretroviral drugs were unavailable at the time. For our first aim, we examined in-utero exposure to HIV and included studies comparing HEU children with HU children. For our second aim, we investigated exposure to maternal ART (defined as at least one antiretroviral drug in pregnancy) and included studies comparing HEU children exposed to different ART regimens, classes, or drugs, or no treatment; we did not require these studies to have a comparison group of HU children. The coprimary outcomes were cognitive development, receptive language, expressive language, fine motor and gross motor development, and social-emotional and adaptive behaviour. Secondary outcomes were head circumference and brain structure. All study designs (interventional, and observational cohort, longitudinal, and cross-sectional) in English or Spanish were included. We excluded conference and poster abstracts.

EW and CJW independently screened all titles, abstracts, and full texts for eligibility. Differences were resolved by discussion with a third reviewer (KAD or AJP). Where relevant, authors were contacted to clarify study eligibility. Search results were de-duplicated in EndNote X8. Study quality and risk of bias of studies comparing neurodevelopment of HEU children and HU children were assessed independently by two authors (CB-C and CJW) using the validated National Heart, Lung, and Blood Institute's Quality Assessment Tool for Observational Cohort and Cross-Sectional Studies.^17^ We adapted the assessment similarly to a previous review^18^ (appendix p 8). Studies were given an overall quality rating of good, fair, or poor as recommended by COSMOS guidance,^15^ based on low, medium, or high risk of bias, respectively.

This systematic review and meta-analysis followed the PRISMA guidelines.^19^ The protocol was registered on PROSPERO (CRD42018075910; appendix p 2).

### Data extraction

Data extraction was done in duplicate (by CJW, EW, and CB-C), including study design and setting, population demographics, exposure (HIV testing and ART exposure), methodology, outcome measures, and results. Outcomes of HEU children were classified by comparison with HU children as better, worse, or no difference on the basis of the individual study significance testing (p<0·05) or absolute or relative differences with confidence intervals in cases where p values were not shown.^20^ Studies comparing different maternal ART regimens were classified with the same method, with one of the ART regimens selected as the reference group. For papers in the meta-analysis, aggregate mean scores with SDs of each neurodevelopmental domain for HEU children and HU children groups were extracted from individual studies. Where mean scores and SDs were not given or the neurodevelopmental outcomes did not fall within the specified domain groupings, we contacted authors for further information.

### Data analysis

Our first aim was to investigate the effect of intrauterine HIV exposure. We did a narrative synthesis of coprimary outcomes compared between HEU children and HU children. We assessed unadjusted results, since studies adjusted for different confounders, and noted any changes on adjusted analyses where reported. For each study, significant differences in neurodevelopmental scores or proportions of developmental delay between the two groups in each domain were recorded and presented using a similar approach to Prado and colleagues*.*^21^

We did a meta-analysis of outcomes reported by six or more studies. As we anticipated substantial methodological heterogeneity and potential for confounding when assessing neurodevelopment, we limited this to high-quality studies and used a random-effects model. Due to the relatively wide age range in some studies, we used age-standardised values, where reported, and did sensitivity analyses using raw scores given the concern that the norms might overestimate development.^22^ Given assessment tools differed across studies, we calculated weighted effect sizes (standardised mean differences with Hedge's correction; mean of HEU children minus mean of HU children, then divided by the pooled SD) with 95% CIs using the group mean and SD for each neurodevelopmental domain. Heterogeneity was estimated with the *I*^2^ test, and Q-values were used to test for between-group differences. We planned to construct a funnel plot to examine publication biases where there were ten or more studies. Statistical significance was set at p<0·05.

For our second aim, we examined associations between maternal ART exposures, stratified by regimen, drug class and individual drugs (where available), and neurodevelopmental domains. We report a synthesis of results in a similar format to the primary aim. If ART studies were sufficiently similar, we planned a second meta-analysis. Finally, we reported a narrative synthesis of the effects of HIV and ART on head circumference and neuroimaging. We used Stata 16.1 for analyses and to derive forest plots.

### Role of the funding source

The funder had no role in study design, data collection, data analysis, data interpretation, or writing of the report.

## Results

**Figure 1:**
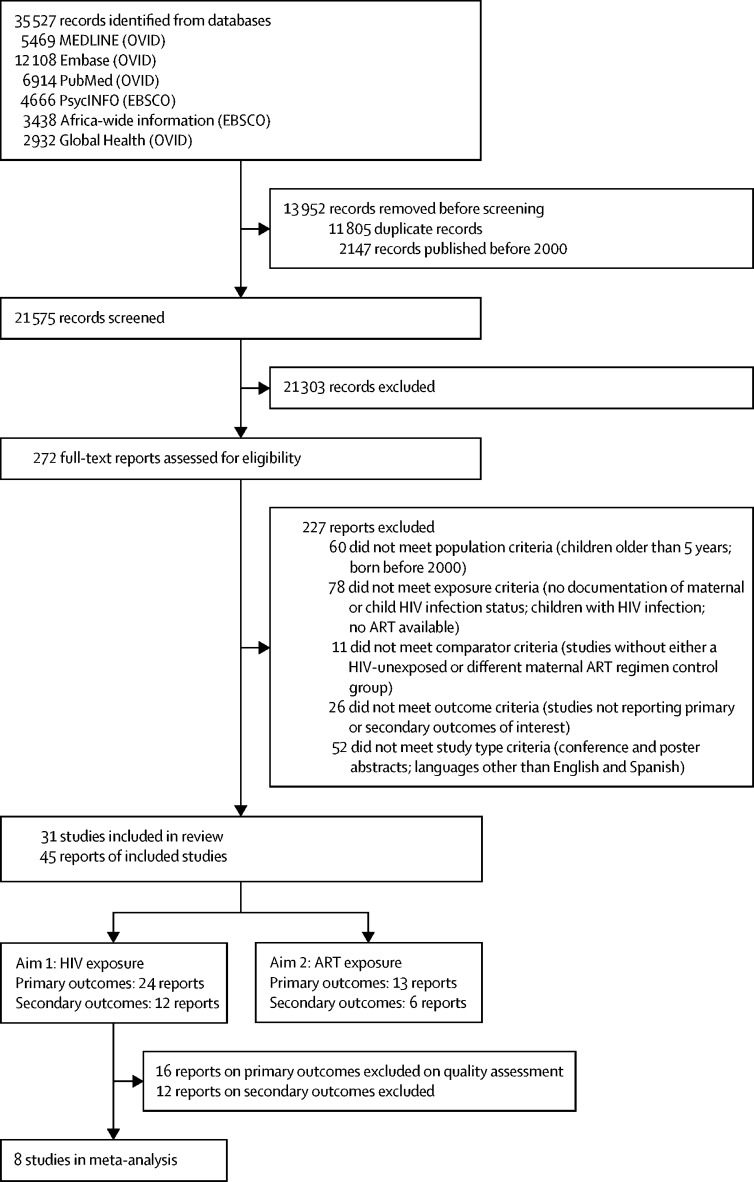
Study selection At the record screening stage, the main categories for excluding records were: (1) population: age range over 5 years; (2) exposure: not examining HIV-exposed uninfected children or only reporting on children living with HIV; and (3) outcome: no neurodevelopment outcomes. Of the total number of reports included (n=45), five contributed results for the primary and secondary outcomes of the first aim, one contributed results for the primary and secondary outcomes of the second aim, and four contributed results for the primary outcomes of both aims (appendix p 18). ART=antiretroviral therapy.

A total of 35 527 references were screened (figure 1). We assessed 272 full-text studies, of which 227 did not meet inclusion criteria, most commonly due to failure to classify maternal and child HIV infection status (appendix pp 9–17). A total of 45 records reporting 31 studies were included, 44 in English and one in Spanish. Of these, 24 articles (21 studies) compared neurodevelopment between HEU children and HU children, 13 articles (ten studies) compared different maternal ART regimens, and 18 articles (13 studies) reported head circumference or neuroimaging (figure 1; appendix p 18).

**Figure 2:**
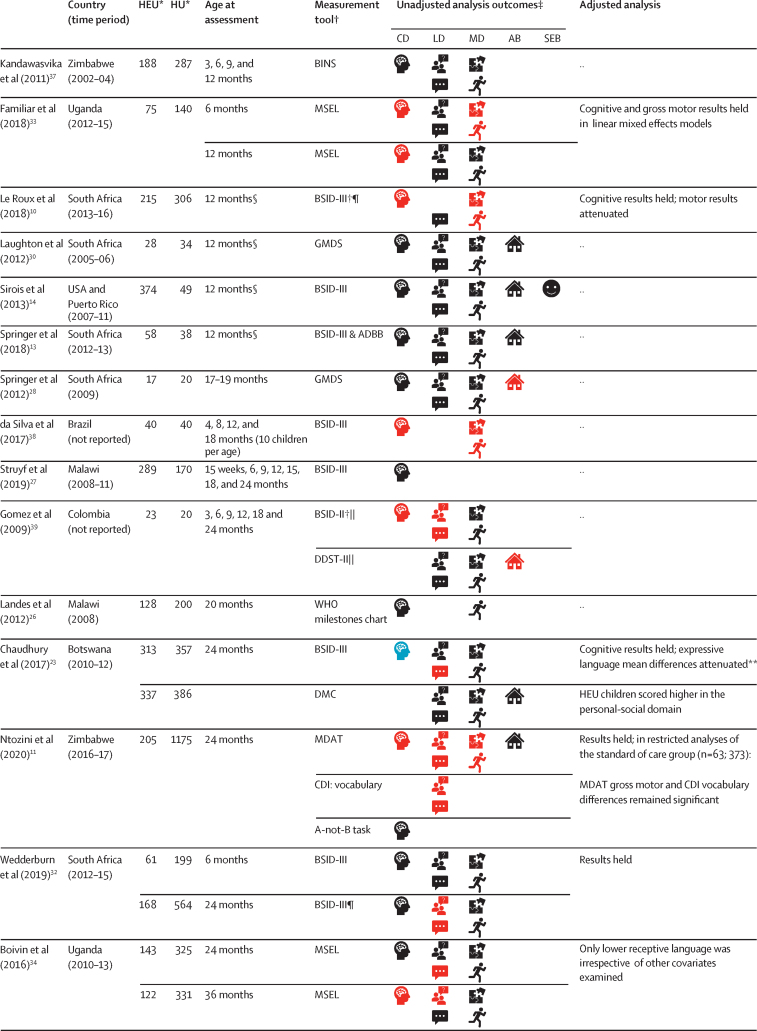

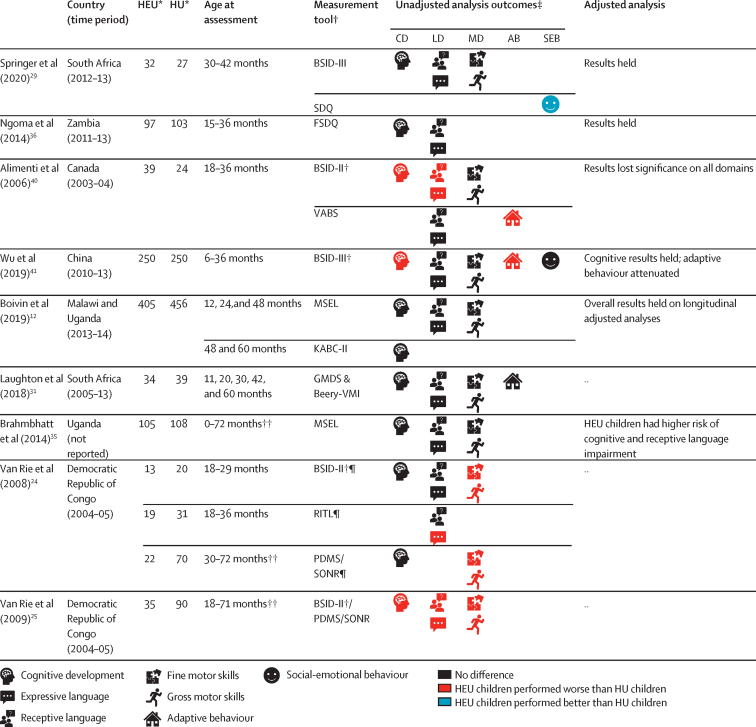
Neurodevelopment of HEU children compared with HU children CD=cognitive development. LD=language development. MD=motor development. AB=adaptive behaviour. SEB=social-emotional behaviour. HEU=HIV-exposed uninfected. HU=HIV-unexposed. ADBB=Alarm Distress Baby Scale. BINS=Bayley Infant Neurodevelopmental Screener. BSID-II=Bayley Scales of Infant and Toddler Development 2nd edition. BSID-III=Bayley Scales of Infant and Toddler Development 3rd edition. CDI=MacArthur-Bates Communicative Development Inventories. DDST-II=Denver Developmental Screening Test 2nd edition. DMC=Developmental Milestones Checklist. FSDQ=Full-Scale Developmental Quotient. GMDS=Griffiths Mental Development Scales. KABC-II=Kaufman Assessment Battery for Children 2nd edition. MDAT=Malawi Developmental Assessment Tool. MSEL=Mullen Scales of Early Learning. PDMS=Peabody Developmental Motor Scales. RITLS=Rossetti Infant-Toddler Language Scale. SDQ=Strengths and Difficulties Questionnaire. SONR=Snijders-Oomen Nonverbal Intelligence Test. VABS=Vineland Adaptive Behaviour Scales. Beery-VMI=Beery Buktenica Test of Visual Motor Integration. *Where the number differed across domains, the highest number is listed. †Where BSID-III composite scores are reported for language and motor development or BSID-II mental development index was used to reflect cognitive and language development; separately, where applicable, cognitive development was assessed using the MSEL cognitive composite score, MDAT total score, or GMDS general quotient. ‡Unadjusted analysis outcomes defined by statistical significance of p<0·05 or through 95% CIs in group comparisons of the mean or comparison of delay where applicable. §Age is given as 12 months if median age of assessment fell within 1 month of this time-point (appendix pp 19–20). ¶Delay reported here; on analysis of mean scores, Le Roux and colleagues^10^ reported no significant group differences in mean scores; Wedderburn and colleagues^32^ reported HEU children had lower receptive and expressive language scores than HU children in both unadjusted and adjusted analyses, and lower cognitive scores on unadjusted analysis. ||BSID-II differences at 6 and 18 months only, DDST differences at 6 months. **On analysis of adverse outcomes, HEU children had significantly more expressive language adverse outcomes than HU children on unadjusted and adjusted analyses. ††Studies included as median age within age range.

For the first aim, comparing neurodevelopment of HEU children versus HU children, 19 (79%) of 24 articles were from Africa,^10^, ^11^, ^12^, ^13^, ^23^, ^24^, ^25^, ^26^, ^27^, ^28^, ^29^, ^30^, ^31^, ^32^, ^33^, ^34^, ^35^, ^36^, ^37^ two (8%) from South America,^38^, ^39^ two (8%) from North America,^14^, ^40^ and one (4%) from Asia.^41^ Characteristics of the studies are detailed in figure 2 and the appendix (pp 19–20). The Bayley Scales of Infant and Toddler Development was the most common measurement tool (11 studies), followed by the Mullen Scales of Early Learning (four studies). All tools are listed in the appendix (p 21). In 12 (57%) of 21 studies, most HEU children were exposed to triple maternal ART (defined as at least three antiretroviral drugs during pregnancy).

Methodological quality varied, with eight reports judged good quality, 11 fair, and five poor (appendix p 22). Sample sizes ranged from 37 to 1380, with only 13 reports including over 50 children per group. The most common methodological concerns were selection bias and loss to follow-up. Blinded outcome assessments were described in eight (33%) of 24 reports. Of the 12 (50%) of 24 reports with adjusted analyses, covariates varied widely (appendix pp 19–20). The high-quality reports had representative study populations, included controls from the same community, and used validated outcome assessments.

Figure 2 shows the synthesis of results from all 24 reports. Two studies from South Africa^13^, ^29^, ^30^, ^31^ and one from Democratic Republic of the Congo^24^, ^25^ provided two reports each. The results were similar at each timepoint in all three studies; therefore, our summary statistics only include each study once. Overall, 12 (57%) of 21 studies reported poorer neurodevelopmental outcomes in HEU children than in HU children in at least one domain on unadjusted analyses, eight (38%) reported no differences in neurodevelopment, and one (5%) found only better social-emotional development in HEU children versus HU children. In the 12 studies reporting adjusted analyses, most results remained unchanged; however, in one Canadian study reporting lower neurodevelopmental scores among HEU children, findings were attenuated after accounting for maternal substance use.^40^

**Figure 3:**
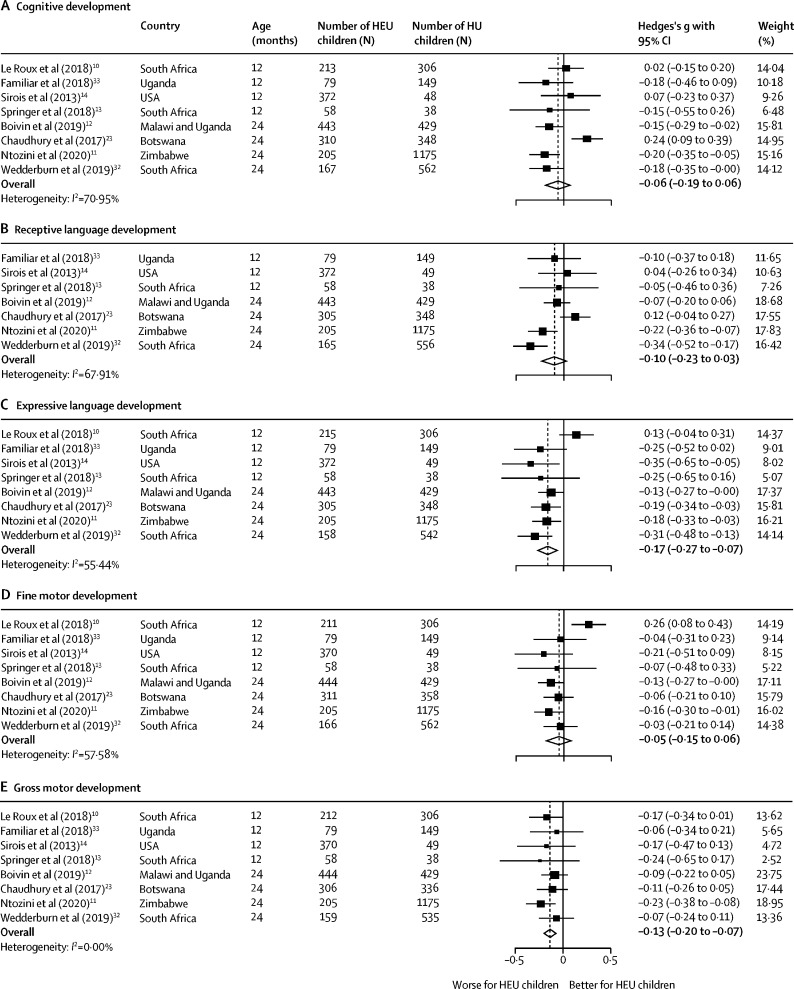
Forest plots of neurodevelopmental outcomes of HEU children compared with HU children included in the meta-analysis HEU=HIV-exposed uninfected. HU=HIV-unexposed.

Among the eight studies^10^, ^11^, ^12^, ^13^, ^14^, ^23^, ^32^, ^33^ eligible for meta-analysis, a slightly higher proportion of studies (five [63%] of eight) reported poorer outcomes across neurodevelopmental domains in HEU children. A total of 1856 HEU children and 3067 HU children from studies in Uganda (n=1), South Africa (n=3), USA and Puerto Rico (n=1), Botswana (n=1), Zimbabwe (n=1), and Malawi plus Uganda (n=1) were included. In all studies except one,^23^ most mothers were taking triple ART during pregnancy; of the HEU children with available data, 1709 (99%) of 1732 had known ART exposure. Among children with regimen data, 1241 (75%) of 1661 were exposed to triple therapy and 414 (25%) of 1661 to zidovudine monotherapy. Forest plots are shown separately for the five neurodevelopmental domains reported in sufficient studies in figure 3. Since most studies reported outcomes at either age 12 or 24 months, we combined these and then did stratified sensitivity analyses. Only Boivin and colleagues^12^ had observations at older ages; however, for consistency we only included their 24-month data.

Overall, HEU children had worse expressive language outcomes (effect size –0·17 [95% CI –0·27 to –0·07], p=0·0013) and gross motor outcomes (–0·13 [–0·20 to –0·07], p<0·0001) compared with HU children, but similar cognitive development (–0·06 [–0·19, 0·06], p=0·34), receptive language development (–0·10 [–0·23 to 0·03], p=0·14), and fine motor development (–0·05 [–0·15 to 0·06], p=0·36). There was moderate heterogeneity in expressive language outcomes (*I*^2^=55·44%, p=0·028) and fine motor outcomes (*I*^2^=57·58%, p=0·021), high heterogeneity in cognitive outcomes (*I*^2^=70·95%, p=0·0011) and receptive language outcomes (*I*^2^=67·91%, p=0·0047), and no heterogeneity in gross motor outcomes (*I*^2^=0%, p=0·85). Sensitivity analyses excluding the one study from a high-income country,^14^ or using aggregate mean raw scores, where available, instead of standardised scores, resulted in similar estimates. Post hoc, on age stratification (age 12 and 24 months), expressive language differences were only apparent at 24 months, which reduced the heterogeneity (12 month effect size –0·16 [–0·42 to 0·11], p=0·24; heterogeneity *I*^2^=72·23%, p=0·013, versus 24 month –0·19 [–0·27 to –0·11], p<0·0001; heterogeneity *I*^2^=0%, p=0·51), whereas gross motor outcomes showed similar findings at both ages.

**Figure 4:**
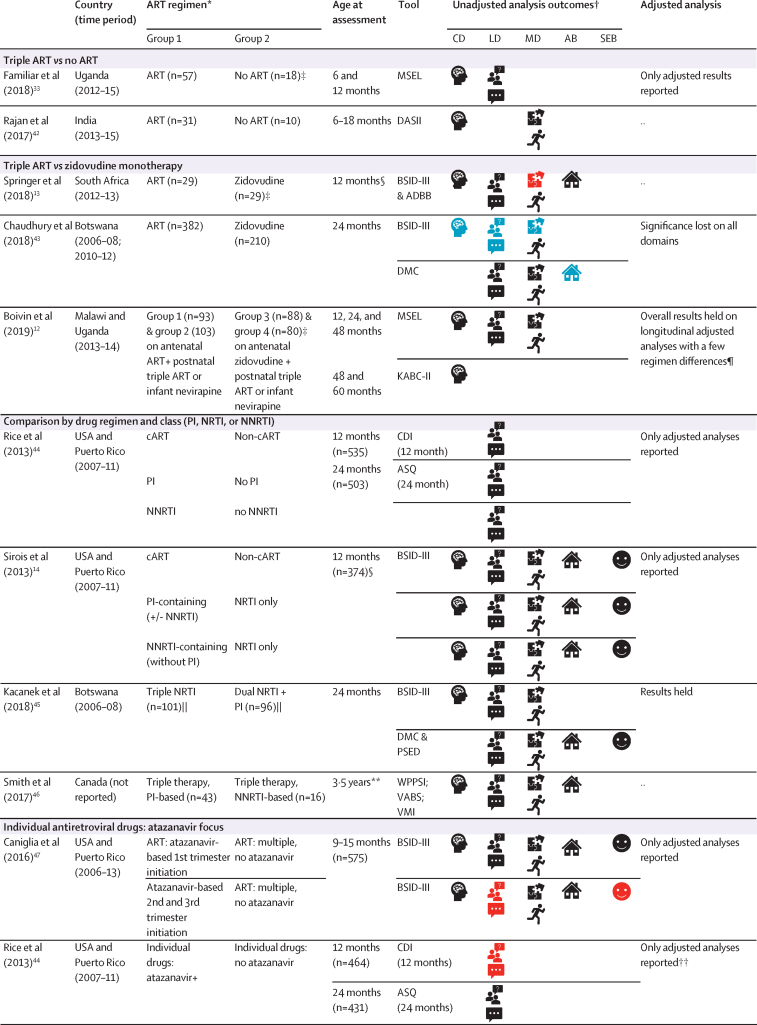

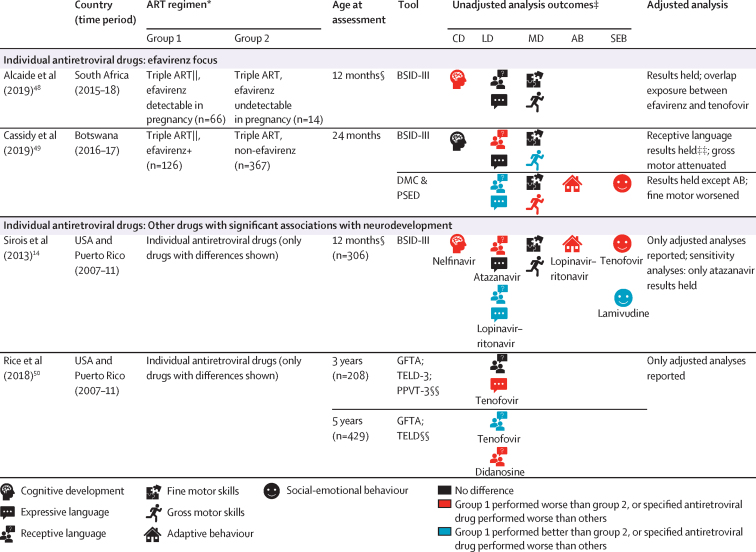
Differences in neurodevelopment of HIV-exposed uninfected children by maternal ART CD=cognitive development. LD=language development. MD=motor development. AB=adaptive behaviour. SEB=social-emotional behaviour. HEU=HIV-exposed uninfected. HU=HIV-unexposed. ART=antiretroviral therapy. NNRTI=non-nucleoside reverse transcriptase inhibitor. NRTI=nucleoside reverse transcriptase inhibitor. PI=protease inhibitor. ASQ=Ages & Stages Questionnaire. BSID-III=Bayley Scales of Infant & Toddler Development 3rd edition. CDI=MacArthur-Bates Communicative Development Inventories. DASII=Development Assessment Scale for Indian Infants. DMC=Developmental Milestones Checklist. GFTA=Goldman-Fristoe Test of Articulation. KABC-II=Kaufman Assessment Battery for Children 2nd edition. MSEL=Mullen Scales of Early Learning. PPVT-3=Peabody Picture Vocabulary Test 3rd edition. PSED=Personal, Social and Emotional Development. TELD-3=Test of Early Language Development 3rd edition. VABS=Vineland Adaptive Behaviour Scales. VMI=Visual Motor Integration. WPPSI=Wechsler Preschool and Primary Scale of Intelligence. cART=combination ART defined in the SMARTT cohort as three or more drugs from two or more antiretroviral classes. *Number (n) given refers to the first visit in studies with multiple time-points, unless otherwise stated; group numbers differ across domains and ages and where multiple different drugs were assessed. †Unadjusted analysis outcomes defined by statistical significance of p<0·05 or through 95% CIs in group comparisons of the mean or comparison of delay where applicable. Where unadjusted analyses were not reported, adjusted analyses are presented instead. ‡These studies also had HU child groups; see figure 2. §Age is given as 12 months if median age of assessment fell within 1 month of this timepoint (appendix pp 23–24). ¶At age 4 years, MSEL cognitive composite scores were higher for children of mothers on antenatal and postnatal triple ART versus children of mothers not on triple ART consistently. ||Kacanek and colleagues^45^ regimen: abacavir/zidovudine/lamivudine versus lopinavir–ritonavir/zidovudine/lamivudine; Alcaide and colleagues^48^ and Cassidy and colleagues^49^ efavirenz regimens: efavirenz/tenofovir/emtricitabine. **Stratified results for the age-point over 5 years are not presented due to review inclusion criteria. ††Multiple individual drugs assessed. At age 12 months, atazanavir increased odds of late language emergence (especially started in 2nd and 3rd trimester). Saquinavir had a similar effect although significance was lost on sensitivity analyses. Other drugs did not have significant associations. ‡‡Conception and 1st trimester efavirenz exposure worse that 2nd and 3rd trimester. §§Language impairment assigned as receptive language; speech impairment assigned as expressive language.

For the second aim, we included 13 articles (10 studies)^12^, ^13^, ^14^, ^33^, ^42^, ^43^, ^44^, ^45^, ^46^, ^47^, ^48^, ^49^, ^50^ examining the effect of maternal ART regimens on child neurodevelopment (figure 4; appendix pp 23–24). Of these, four articles were from the Surveillance Monitoring for ART Toxicities (SMARTT) protocol of the Pediatric HIV/AIDS Cohort Study. Most studies used a cohort design; only two studies randomised ART regimens.^12^, ^45^ We were unable to do a meta-analysis given the regimen heterogeneity across studies.

Small studies comparing neurodevelopment of HEU children exposed to triple ART versus no ART in the first 24 months found similar outcomes^33^, ^42^ as did larger studies examining triple ART versus monotherapy from ages 1–5 years^13^, ^43^, ^12^ (figure 4). Although one study in Botswana reported better neurodevelopment with triple ART compared with zidovudine only, this attenuated after adjustment for confounders.^43^ Reports from the SMARTT cohort showed no evidence of differences in neurodevelopment at ages 12 or 24 months between children exposed to combination ART (defined as at least three drugs from at least two different drug classes) and non-combination ART regimens (including three nucleoside reverse transcriptase inhibitors [NRTIs], one or two drugs, or no ART);^14^, ^44^ protease inhibitor-containing or non-nucleoside reverse transcriptase inhibitor-containing (NNRTI) regimens compared with NRTI-only regimens;^14^ protease inhibitor-containing versus no protease inhibitors and NNRTI-containing versus no NNRTIs;^44^ and triple NRTI versus combination ART regimens.^50^ Similarly, a randomised study from Botswana reported no neurodevelopmental differences comparing triple NRTI with dual NRTI plus protease inhibitor regimens at 24 months.^45^

Analyses of individual drugs were predominantly reported from the SMARTT cohort; of the multiple drugs assessed, most had no evidence of significant associations with neurodevelopment. However, there was a signal for worse language outcomes in children exposed to atazanavir-containing versus non-atazanavir regimens at age 12 months,^14^ particularly when initiated in the second or third trimester,^47^ although this was no longer apparent at age 24 months.^44^ The Tshipidi-plus study from Botswana found HEU children who were exposed to efavirenz-containing regimens had lower performance-rated receptive language on the Bayley Scales of Infant and Toddler Development third edition at age 24 months compared with non-efavirenz-containing regimens. However, caregiver-rated language scores were higher in the efavirenz group.^49^

In the analysis of secondary outcomes (table 1; appendix pp 25–26), 11 reports from ten studies compared head circumference between HEU children and HU children.^13^, ^28^, ^29^, ^30^, ^39^, ^51^, ^52^, ^53^, ^54^, ^55^, ^56^ Results were heterogeneous; five of ten studies found no difference between the two groups at ages 0–36 months.^13^, ^28^, ^29^, ^30^, ^51^, ^53^ One small study found a difference among neonates but not at later ages.^39^ Beyond the neonatal period, the four largest studies (≥400 children each; ages 0–24 months), all from Africa, found that HEU children had significantly lower Z scores than HU children,^52^, ^54^, ^55^, ^56^ which held on adjusted analyses. Two reports found no relationships between combination ART regimens and head circumference (table 2);^57^, ^58^ however, in the SMARTT cohort, tenofovir^59^ and atazanavir^47^ were associated with smaller head circumference at age 1 year but not at age 2 years,^60^ whereas efavirenz was associated with microcephaly, and poorer neurodevelopment in children with microcephaly in the first 5 years.^61^ Estimates of microcephaly varied from 1% of HEU children^52^ to 7·5%.^57^ Only one neuroimaging study in South Africa compared HEU neonates and HU neonates: brain microstructural differences were identified, along with correlations between white matter microstructure and neurobehaviour.^62^

**Table 1:** Head circumference and neuroimaging outcomes of HEU children compared with HU children (aim 1)

	**Country (time period)**	HEU children*	HU children*	**Assessment by age**†	**Adjusted analyses and comments**
**Head circumference of HEU children *vs* HU children**					
Donald et al (2017)^51^	South Africa (2012–15)	131	536	No effect at birth	Results held on adjusted analysis
Filteau et al (2011)^55^	Zambia (2005–09)	125	382	HEU children had smaller head circumference at 6 months	Baseline trial results reported
Le Roux et al (2019)^52^	South Africa (2013–16)	461	411	HEU children had smaller head circumference at birth, 3 months, 9 months, and 12 months; no effect at 6 months	Results held on adjusted analysis; at 12 months 1% of HEU children had microcephaly and 17% had macrocephaly; of HU children 1% had microcephaly and 22% macrocephaly
Laughton et al (2012)^30^	South Africa (2005–06)	28	34	No effect at 12 months	..
Neri et al (2013)^53^	USA (2006–09)	82	82	No effect at average age 10 months (age range was 2 weeks to 2 years)	Results held on adjusted analysis
Springer et al (2018)^13^	South Africa (2012–13)	58	38	No effect at 12 months	
Jumare et al (2019)^54^	Nigeria (2013–17)	297	103	HEU children had smaller head circumference from birth to 18 months	Longitudinal analyses; lower head circumference-for-age Z score results held on adjusted analysis
Gomez et al (2009)^39^	Colombia (not reported)	23	20	HEU children had smaller head circumference at birth; no effect at 3, 6, 9, 12, and 24 months	..
Aizire et al (2020)^56^	Malawi and Uganda (2013–14)	471	462	No effect at 12 months; HEU children had smaller head circumference at 2 years in Uganda; no effect at 2 years in Malawi	Results held on adjusted analysis; risk of head circumference-for-age Z score less than WHO median increased among HEU children *vs* HU children at 24 months
Springer et al (2012)^28^	South Africa (2009)	17	20	No effect at 17–19 months	..
Springer et al (2020)^29^	South Africa (2012–13)	32	27	No effect at 30–42 months	4 (12·5%) HEU children had macrocephaly
**Neuroimaging of HEU children *vs* HU children**					
Tran et al (2016)^62^	South Africa (2012–15)	15	22	HEU children had altered neuroimaging findings at birth compared with HU children	Diffusion tensor imaging; altered white matter microstructure showing higher fractional anisotropy in the middle cerebellar peduncles of HEU children compared with HU children on adjusted analyses; higher fractional anisotropy in the left uncinate fasciculus correlated with abnormal neurological scores of HEU children

HEU=HIV-exposed uninfected. HU=HIV-unexposed.

* Number (n) given refers to the first visit in studies with multiple timepoints.

† Assessment by age defined by statistical significance of p<0·05 or through 95% CIs in group comparisons of the mean or comparison of delay using dichotomised variables.

**Table 2:** Head circumference by different maternal ART (aim 2)

	**Country (time period)**	Group A*	Group B*	**Assessment by age**†	**Adjusted analyses and comments**
Spaulding et al (2016)^57^	Latin America and Caribbean (2002–09)	Multiple ART combinations (n=1400)	..	No effect on head circumference at 6–12 weeks and 6 months	Microcephaly and neurological conditions assessed; no difference on timing of initiation of combination ART or specific drugs; microcephaly reported in 7·5% of HEU children
Pintye et al (2015)^58^	Kenya (2013)	Triple ART with tenofovir (n=51)	Triple ART without tenofovir (n=104)	No effect on head circumference at 6 weeks and 9 months	No associations between prenatal tenofovir use and head circumference-for-age Z score in 6-week or 9-month infant cohorts.
Siberry et al (2012)^59^	USA and Puerto Rico (2005–10)	Triple ART with tenofovir (n=274)	Triple ART without tenofovir (n=416)	No effect on head circumference at birth; tenofovir associated with smaller head circumference at 12 months	Results held on adjusted analysis
Caniglia et al (2016)^47^	USA and Puerto Rico (2006–13)	ART with atazanavir (n=127)	ART without atazanavir (n=525)	Atazanavir associated with smaller head circumference at 12 months	Results held on adjusted analysis; overlap between atazanavir and tenofovir in regimens
Jacobson et al (2017)^60^	USA and Puerto Rico (2007–11)	Triple ART, multiple drugs (n=509)	..	No effect on head circumference at 2 years	No difference by ART regimen or timing of initiation on unadjusted or adjusted analyses; compared tenofovir, atazanavir, nelfinavir, and boosted protease inhibitor regimens
Williams et al (2020)^61^	USA and Puerto Rico (2007–17)	Individual drugs (n=3055); ART with efavirenz (n=141)	ART without efavirenz (n=2842)	Efavirenz associated with smaller head circumference; microcephaly assessed	Efavirenz exposure was associated with increased risk of microcephaly on adjusted analysis; no difference preconception or postconception initiation; more pronounced associations with efavirenz regimens containing zidovudine plus lamivudine compared to tenofovir plus emtricitabine; protective associations with darunavir; increased risk with fosamprenavir; microcephaly was associated with worse neurodevelopment in all domains; multiple drugs assessed and efavirenz was the association reported that survived in the fully adjusted model

ART=antiretroviral therapy. HEU=HIV-exposed uninfected.

* Number (n) given refers to the first visit in studies with multiple timepoints.

† Assessment by age defined by statistical significance of p<0·05 or through 95% CIs in group comparisons of the mean or comparison of delay using dichotomised variables.

## Discussion

We systematically reviewed neurodevelopment in HEU children aged 0–5 years and identified 45 reports from 31 studies across four continents; most studies were from sub-Saharan Africa, where the majority of HEU children live. Although findings were heterogeneous, over half of all studies reported poorer neurodevelopment in HEU children than in HU children in at least one domain. Variability in study design, quality, population characteristics, and assessment tools might explain differences in results across studies. Among high-quality studies, in which 99% of HEU children were exposed to maternal ART, there was evidence that HEU children have poorer expressive language and gross motor function than HU children, although the deficits are subtle with relatively small effect sizes. Timing of assessment appears to affect findings, with language deficits becoming evident after age 12 months, highlighting the importance of long-term follow-up. We found no evidence of consistent associations between specific ART regimens and neurodevelopment, although generalisability of the evidence is limited.

Our findings of impaired expressive language and gross motor development in HEU children are consistent with studies from before the introduction of ART.^5^ Language problems have long been recognised in children with HIV,^63^ with language expression more affected than comprehension.^24^, ^64^ Furthermore, a previous meta-analysis in HEU children including studies from before ART was available found motor function was affected.^7^ Therefore, despite reduced maternal morbidity and mortality due to ART, the negative effects of HIV exposure on language and motor skills remain. Expressive language and gross motor function are measured less often at older ages,^65^ representing an important research gap. However, one US study identified language problems into adolescence, suggesting impairments might persist.^66^ Given that early language predicts school performance,^67^, ^68^ and early motor skills influence other facets of development,^69^ longitudinal follow-up is crucial.

There was no evidence of cognitive impairment in the meta-analysis, consistent with studies reporting similar cognitive scores between HEU children and HU children at age 6–11 years.^65^ Although another meta-analysis reported that cognitive domains are affected in young HEU children,^7^ this assessed the mental development index which combines cognitive and language development; our findings show the importance of separating individual neurodevelopmental domains. Even within our analyses there was high heterogeneity across assessments of the cognitive domain. Furthermore, the restricted analysis of high-quality studies in their meta-analysis^7^ showed no cognitive differences between HEU children and HU children, portraying the importance of focusing on studies with low bias. Social-emotional and adaptive behaviour, defined as living skills that enable everyday function, did not differ between the two groups in high-quality studies in our review. However, the low number of studies reporting on these outcomes prevent reliable conclusions being drawn; further investigation is needed.

WHO guidelines changed in 2012 to recommend universal triple ART for pregnant and breastfeeding women (termed Option B+). To our knowledge, this is the first systematic review to assess the effect of specific ART exposure on the neurodevelopment of HEU children. Overall, our results are reassuring, showing no clear evidence of associations between different ART regimens and drug classes assessed (ie, triple therapy, monotherapy, NRTI, NNRTI, or protease inhibitor-based) and neurodevelopment. However, interpretation of these results is limited by small study numbers and heterogeneous comparison groups. Data on individual drugs are dominated by publications from the observational SMARTT study, which includes children exposed to multiple different combinations. Overall, findings from SMARTT are encouraging,^70^ albeit with some concerns regarding efavirenz^61^ and atazanavir.^47^ However, given the non-randomised design in a US population, it is unclear how generalisable these findings are. An observational study from Botswana reported conflicting results for efavirenz exposure and language,^49^ suggesting further evaluation is needed, with ongoing pharmacovigilance to monitor new drugs.

Secondary outcomes included head circumference, which is often used as a surrogate marker of brain growth and development and might serve as a useful biomarker in clinical practice. Our findings were mixed, and the prevalence of microcephaly varied across studies, suggesting that more robust evidence is needed with long-term follow-up. However, larger studies did suggest a small reduction in head circumference in HEU children compared with HU children. There was no evident association between head circumference and specific ART regimens or drug classes; however, potential associations with efavirenz^61^ were reported from the SMARTT cohort. The SMARTT study also found a relationship between microcephaly in HEU children and neurodevelopmental impairment, which needs to be explored further. Only one neonatal study included neuroimaging and identified white matter alterations associated with adverse neurodevelopment in HEU children compared with HU children, suggesting a potential neurological pathway,^62^ which paves the way for further work. We did not specifically evaluate mechanisms in our review, but others have discussed this in detail.^71^

Our study had several strengths. We reviewed multiple neurodevelopmental outcomes among HEU children up to age 5 years, from diverse contexts across several continents. The meta-analysis addressed sample size and methodological weaknesses of individual studies. We used effect sizes to combine measurement tools, allowing us to identify vulnerability in specific domains and to estimate a measure of effect that could be applied to different tools and ages in the future. Health-care workers can be guided by the effect sizes and examine for small delays in development in this group of children. Our study builds on previous reviews^5^, ^6^, ^7^ with larger, higher-quality African studies included in the meta-analysis; furthermore, we specifically focused on ART and neuroimaging. Several limitations and research gaps were identified (panel). There was substantial heterogeneity across study designs, sample sizes, measurement tools, blinding, quality, and population demographics; only half of the studies included adjusted analyses, and confounder selection varied. Other contributors to child development were not assessed, including vision and hearing loss, which has been reported in older HEU children.^72^ Given the heterogeneity in studies, the results of the meta-analysis should be treated with caution and a causal relationship should not be assumed due to the potential for confounding. However, combining studies with low risk of bias is acceptable, even in the presence of statistical heterogeneity.^15^ We used random-effects models, and combined results across different tools, which is appropriate in the early years (age 0–3 years) in which neurodevelopment has more global commonalities in skill development than in later years.^73^, ^74^ Evidence is scarce for the analysis of ART. Results for individual antiretroviral drugs were predominantly from one US observational study, which limits generalisability. Due to a paucity of study reporting, we were unable to assess the effect of timing of maternal ART initiation that might have influenced infant outcomes. Only two studies randomised ART use,^12^, ^45^ and newer drugs including integrase inhibitors were not assessed.^75^ The reliance on observational data provides several challenges in interpretation. Studies had difficulty separating specific drugs, and ART comparison groups often comprise multiple different antiretroviral combinations. Furthermore, maternal ART initiation might be a proxy for HIV disease severity, and mothers might change regimens during pregnancy.

Panel **Methodological considerations for future studies examining the neurodevelopment of HEU children**

Given the known effect of multiple factors on neurodevelopment, a more coherent approach is needed in which a unified set of covariates are measured and reported with adequate comparator groups to ensure consistency across studies and allow for generalisability.

A standardised framework for assessments with validated cross-cultural measurements is required to improve comparability between different study settings with contextually appropriate norms. Reporting categorical delay scores is useful from a clinical perspective; however, the selection of the threshold is often arbitrary and might miss capturing the full relationship. Therefore, reporting both continuous and categorical measures in tandem might be the best approach.

Individual neurodevelopmental domains should be examined separately. The use of multiple tools for assessing the different components of domains should be considered. This is particularly relevant given the language deficits identified in HEU children, since multiple indicators for speech and language might differentiate between vocabulary, grammar, and speech. As children grow older, differentiating between the various components of executive function also becomes increasingly important.

The definitions of HEU and HIV-unexposed need to be carefully documented. We had to exclude many studies that combined HEU children and children with HIV infection together as HIV-exposed, or which combined children with and without HIV exposure together as HIV-uninfected. In the first instance, differences are likely to be overestimated due to the inclusion of children with HIV, and in the latter, differences might be underestimated due to the inclusion of children without HIV exposure.

Studies should consider factors that are known to affect child neurodevelopment at the design stage (population criteria or covariates), such as low birthweight and preterm birth, small-for-gestational age, hearing impairment, genetic syndromes, and neurological disorders.

Ongoing ART surveillance is needed and randomised controlled trials might help to identify the ART regimens that lead to optimal outcomes. This requires examination of duration of exposure (including ART exposure antenatally and postnatally through breastfeeding, and child prophylaxis) with adequate comparison groups of specific regimens.

Given the dynamic nature of neurodevelopment, the developmental trajectory of HEU children compared with their unexposed peers should form the basis for future work. More large-scale longitudinal studies are needed given the potential subtle deficits early in life, and follow-up is required to determine longer-term effects.

ART=antiretroviral therapy. HEU=HIV-exposed uninfected.

In conclusion, early-life neurodevelopment of HEU children is modestly impaired, specifically in expressive language and gross motor domains. Although effect sizes were small, our findings suggest a subtle yet clear demarcation of differences in abilities between HEU children and HU children at young ages. Given the growth in the global population of HEU children, with the largest increase in Africa, this difference in neurodevelopmental function might have a substantial effect together with other risks faced by this population. There were no consistent signals that specific ART regimens or drug classes affect neurodevelopment; however, evidence is scarce and well designed randomised trials are required. Understanding the potential toxicities or relative neuroprotection of antiretrovirals would allow modification of maternal ART regimens to optimise infant neurodevelopment. There was a scarcity of neuroimaging studies, and our review raises some important methodological considerations for future studies examining neurodevelopment in HEU children (panel). Greater understanding of neurodevelopment in this population will aid identification of vulnerable infants to allow early prevention and intervention strategies during developmentally sensitive periods. The apparent detriment in early motor and language skills among these children calls for more longitudinal studies to investigate neurodevelopmental trajectories, delineate mechanisms, and inform recommendations to support this growing population.

## Data sharing

The study protocol and materials are available at https://osf.io/yeqj3/ and in the appendix (pp 2–26).

## Declaration of interests

AJP declares paid participation on the Botnar Research Centre for Child Health independent external review board and is a member of several data and safety monitoring boards with no payment, none of which relate to the current research. DJS has received research grants or consultancy honoraria from Discovery, Johnson & Johnson, Lundbeck, Sanofi, Servier, Takeda, and Vistagen. All other authors declare no competing interests.
