# Supplementary material for: Early neurodevelopment of HIV-exposed uninfected children in the era of antiretroviral therapy: a systematic review and meta-analysis
Source: Lancet Child Adolesc Health. 2022 Jun;6(6):393–408. doi: 10.1016/S2352-4642(22)00071-2 (PMC9090907; doi:10.1016/S2352-4642(22)00071-2)
Supplement: Supplementary appendix [file mmc1.pdf]

THE LANCET
Child & Adolescent Health

Supplementary appendix

This appendix formed part of the original submission and has been peer reviewed. We post it as supplied by the authors.

Supplement to: Wedderburn CJ, Weldon E, Bertran-Cobo C, et al. Early neurodevelopment of HIV-exposed uninfected children in the era of antiretroviral therapy: a systematic review and meta-analysis. *Lancet Child Adolesc Health* 2022; published online April 25. https://doi.org/10.1016/ S2352-4642(22)00071-2.

Supplementary Information

Early neurodevelopment of HIV-exposed uninfected children in the era of antiretroviral therapy: a systematic review and meta-analysis

Catherine J Wedderburn, Ella Weldon, Cesc Bertran-Cobo, Andrea M Rehman, Dan J Stein, Diana M Gibb, Shunmay Yeung, Andrew J Prendergast,[¶] Kirsten A Donald[¶]

[¶] AJP and KAD are joint senior authors

This PDF file includes:

S1 Appendix PROSPERO systematic review protocol

S2 Appendix Electronic search strategy by database

S3 Appendix PECO inclusion and exclusion criteria

S4 Appendix Quality assessment tool

S5 Appendix Characteristics of studies excluded from full-text review

S6 Appendix List of included studies and relevant articles

S7 Appendix Characteristics of studies examining neurodevelopment of HEU children compared to HU children

S8 Appendix Child development tools used across included studies

S9 Appendix Quality assessment and risk of bias of studies comparing HEU and HU children

S10 Appendix Characteristics of studies of HEU children comparing different maternal ART regimens

S11 Appendix Characteristics of studies examining head circumference and neuroimaging

References

S1 Appendix: PROSPERO systematic review protocol

Link: https://www.crd.york.ac.uk/prospero/display_record.php?ID=CRD42018075910

Protocol deviations:

- Studies including children born before January 2000 or where ART was documented not to be available at the time of the study were excluded. This differed to the original inclusion period of January 1990 for the date of search. We made this change in order to ensure relevance to the current ART era, fitting with the WHO guidelines advocating for PMTCT interventions in the year 2000.
- We were unable to assess vision, hearing, and intellect as outcomes due to a lack of appropriate studies within this age range.
- We used the validated National Heart, Lung, and Blood Institute (NHLBI) Quality Assessment Tool for Observational Cohort and Cross-Sectional Studies (National Heart, Lung, and Blood Institute [NHLBI], 2014) to assess risk of bias instead of the originally specified Newcastle-Ottawa Quality Assessment Scale (NOS). We found this tool had many similarities to the NOS but allowed us to better assess the various aspects of bias for all types of studies included in this review.
- We performed formal risk of bias assessment for all Aim 1 outcomes which went forward into meta-analysis. Following review of the papers for Aim 2, we noted we were unable to perform a meta-analysis for the ART analyses due to study heterogeneity and that the risk of bias tool did not adequately focus on additional areas of concern specific to ART analyses. Therefore, instead of performing individual study risk of bias using a formal tool for these papers we describe the overall limitations and risk of bias specific to studies in Aim 2.
- Pre-specified sensitivity analyses included only studies with adequate comparison groups and excluding those with a moderate or high risk of bias. Due to limited numbers of studies fitting these criteria we did not perform subgroup analyses on breastfeeding. Similarly, we originally planned to stratify by region and socioeconomic status. However, given the limited number of studies, and lack of comparable reporting on socioeconomic status, we were only able to perform a sensitivity analysis of the meta-analysis excluding the one study from a high-income country.

S2 Appendix: Electronic search strategy by database

MEDLINE

1. (infan* or newborn* or new-born* or perinat* or neonat* or baby or babies or toddler* or child* or pediatric* or paediatric*).mp. [mp=title, abstract, original title, name of substance word, subject heading word, keyword heading word, protocol supplementary concept word, rare disease supplementary concept word, unique identifier, synonyms]
2. exp CHILD, PRESCHOOL/
3. exp INFANT/
4. exp Pediatrics/
5. exp INFANT, NEWBORN/
6. 1 or 2 or 3 or 4 or 5
7. (HIV or HIV-1 or HIV-2 or human immunodeficiency virus or human immune deficiency virus or human immune-deficiency virus or HIV infect* or HIV-infected or living with HIV or HIV-positive or AIDS or Acquired Immunodeficiency Syndrome or Acquired Immune Deficiency Syndrome or HEU or HIV-exposed or HIV-exposed-uninfected or HIV-uninfected or HAART or antiretroviral* or anti-retroviral* or anti-HIV agents or HIV treatment or PMTCT or prevention of mother-to-child transmission).mp. [mp=title, abstract, original title, name of substance word, subject heading word, keyword heading word, protocol supplementary concept word, rare disease supplementary concept word, unique identifier, synonyms]
8. exp HIV/ or exp HIV-2/ or exp HIV-1/ or exp HIV INFECTIONS/
9. exp HIV PROTEASE INHIBITORS/ or exp ANTI-HIV AGENTS/ or exp HIV INTEGRASE INHIBITORS/ or exp HIV FUSION INHIBITORS/
10. exp Reverse Transcriptase Inhibitors/
11. exp Anti-Retroviral Agents/ or exp ANTIRETROVIRAL THERAPY, HIGHLY ACTIVE/ or exp Acquired Immunodeficiency Syndrome/
12. 7 or 8 or 9 or 10 or 11
13. (Neurodevelop* or (develop* adj4 child) or neurocog* or cogniti* or communication or language or speech or verbal or motor or neuromotor or locomotor or neuroanatom* or (brain adj4 structure) or (brain adj4 microstructure) or (white adj4 matter) or head circumference or (developmental adj4 delay*) or (developmental adj4 disorder*) or (developmental adj4 disabilit*)).mp. [mp=title, abstract, original title, name of substance word, subject heading word, keyword heading word, protocol supplementary concept word, rare disease supplementary concept word, unique identifier, synonyms]
14. exp CHILD DEVELOPMENT DISORDERS, PERVASIVE/ or exp MUSCULOSKELETAL DEVELOPMENT/ or exp HUMAN DEVELOPMENT/ or exp LANGUAGE DEVELOPMENT DISORDERS/ or exp "MALFORMATIONS OF CORTICAL DEVELOPMENT"/ or exp "MALFORMATIONS OF CORTICAL DEVELOPMENT, GROUP II"/ or exp "GROWTH AND DEVELOPMENT"/ or exp "MALFORMATIONS OF CORTICAL DEVELOPMENT, GROUP I"/ or exp LANGUAGE DEVELOPMENT/ or exp "NATIONAL INSTITUTE OF CHILD HEALTH AND HUMAN DEVELOPMENT (U.S.)"/ or exp CHILD DEVELOPMENT/
15. exp CHILD BEHAVIOR/ or exp CHILD BEHAVIOR DISORDERS/
16. exp DEVELOPMENTAL DISABILITIES/
17. exp NEUROCOGNITIVE DISORDERS/ or exp NEURODEVELOPMENTAL DISORDERS/ or exp COMMUNICATION DISORDERS/
18. exp CHILD LANGUAGE/
19. exp Prenatal Exposure Delayed Effects/
20. 13 or 14 or 15 or 16 or 17 or 18 or 19
21. 6 and 12 and 20

EMBASE search

1. (infan* or newborn* or new-born* or perinat* or neonat* or baby or babies or toddler* or child* or pediatric* or paediatric*).mp. [mp=title, abstract, heading word, drug trade name, original title, device manufacturer, drug manufacturer, device trade name, keyword, floating subheading word, candidate term word]
2. exp preschool child/
3. exp infant/
4. exp pediatrics/
5. exp newborn/
6. 1 or 2 or 3 or 4 or 5

7. (HIV or HIV-1 or HIV-2 or human immunodeficiency virus or human immune deficiency virus or human immune-deficiency virus or HIV infect* or HIV-infected or living with HIV or HIV-positive or AIDS or Acquired Immunodeficiency Syndrome or Acquired Immune Deficiency Syndrome or HEU or HIV-exposed or HIV-exposed-uninfected or HIV-uninfected or HAART or antiretroviral* or anti-retroviral* or anti-HIV agents or HIV treatment or PMTCT or prevention of mother-to-child transmission).mp. [mp=title, abstract, heading word, drug trade name, original title, device manufacturer, drug manufacturer, device trade name, keyword, floating subheading word, candidate term word]
8. exp Human immunodeficiency virus/ or exp Human immunodeficiency virus 2/ or exp Human immunodeficiency virus 1/
9. exp Human immunodeficiency virus infection/ or exp integrase inhibitor/ or exp Human immunodeficiency virus fusion inhibitor/ or exp Human immunodeficiency virus proteinase inhibitor/ or exp anti human immunodeficiency virus agent/ or exp nonnucleoside reverse transcriptase inhibitor/
10. exp antiretrovirus agent/ or exp highly active antiretroviral therapy/ or exp acquired immune deficiency syndrome/ or exp antiretroviral therapy/
11. 7 or 8 or 9 or 10
12. (Neurodevelop* or (develop* adj4 child) or neurocog* or cogniti* or communication or language or speech or verbal or motor or neuromotor or locomotor or neuroanatom* or (brain adj4 structure) or (brain adj4 microstructure) or (white adj4 matter) or head circumference or (developmental adj4 delay*) or (developmental adj4 disorder*) or (developmental adj4 disabilit*)).mp. [mp=title, abstract, heading word, drug trade name, original title, device manufacturer, drug manufacturer, device trade name, keyword, floating subheading word, candidate term word]
13. exp MUSCULOSKELETAL DEVELOPMENT/ or exp developmental language disorder/ or exp LANGUAGE DEVELOPMENT/ or exp child behavior/ or exp behavior disorder/
14. exp cognition/ or exp DEVELOPMENTAL DISORDER/
15. exp child development/
16. 12 or 13 or 14 or 15
17. 6 and 11 and 16

Pubmed search
infan* or newborn* or new-born* or perinat* or neonat* or baby or babies or toddler* or child or children or pediatric* or paediatric*

HIV or HIV-1 or HIV-2 or human immunodeficiency virus or human immune deficiency virus or human immune-deficiency virus or HIV infect* or HIV-infected or “living with HIV” or HIV-positive or AIDS or “Acquired Immunodeficiency Syndrome” or HEU or HIV-exposed or HIV-exposed-uninfected or HIV-uninfected or HAART or antiretroviral* or anti-retroviral* or “anti-HIV agents” or “HIV treatment” or PMTCT or “prevention of mother-to-child transmission”

Neurodevelop* or “child develop*” or neurocog* or cogniti* or communication or language or speech or verbal or motor or neuromotor or locomotor or neuroanatom* or “brain structure” or “brain microstructure” or “white matter” or “head circumference” or “developmental delay*” or “developmental disorder*” or “developmental disability”

1. 1 and 2 and 3

PsycINFO
1. (infan* or newborn* or new-born* or perinat* or neonat* or baby or babies or toddler* or child* or pediatric* or paediatric*).mp. [mp=title, abstract, original title, name of substance word, subject heading word, keyword heading word, protocol supplementary concept word, rare disease supplementary concept word, unique identifier, synonyms]
2. exp preschool students/
3. exp pediatrics/
4. 1 or 2 or 3
5. (HIV or HIV-1 or HIV-2 or human immunodeficiency virus or human immune deficiency virus or human immune-deficiency virus or HIV infect* or HIV-infected or living with HIV or HIV-positive or AIDS or Acquired Immunodeficiency Syndrome or Acquired Immune Deficiency Syndrome or HEU or HIV-exposed or HIV-exposed-uninfected or HIV-uninfected or HAART or antiretroviral* or anti-retroviral* or anti-HIV agents or HIV treatment or PMTCT or prevention of mother-to-child

transmission).mp. [mp=title, abstract, original title, name of substance word, subject heading word, keyword heading word, protocol supplementary concept word, rare disease supplementary concept word, unique identifier, synonyms]

6. exp hiv/ or exp aids/
7. exp antiviral drugs/
8. 5 or 6 or 7
9. (Neurodevelop* or (develop* adj4 child) or neurocog* or cogniti* or communication or language or speech or verbal or motor or neuromotor or locomotor or neuroanatom* or (brain adj4 structure) or (brain adj4 microstructure) or (white adj4 matter) or head circumference or (developmental adj4 delay*) or (developmental adj4 disorder*) or (developmental adj4 disabilit*)).mp. [mp=title, abstract, original title, name of substance word, subject heading word, keyword heading word, protocol supplementary concept word, rare disease supplementary concept word, unique identifier, synonyms]
10. exp childhood development/ or exp early childhood development/ or exp motor development/ or exp psychological development/ or exp psychomotor development
11. exp brain development/
12. exp neonatal development/
13. exp language development/ or exp cognitive development/ or exp language delay/ or exp language disorders/ or exp speech development/
14. exp behavior problems/ or exp behavior/ or exp behavior disorders/
15. exp developmental disabilities/ or exp delayed development/
16. exp neurodevelopmental disorders/
17. exp Infant Development/
18. 9 or 10 or 11 or 12 or 14 or 15 or 16 or 17
19. 4 and 8 and 18

Global Health

1. (infan* or newborn* or new-born* or perinat* or neonat* or baby or babies or toddler* or child* or pediatric* or paediatric*).mp. [mp=title, abstract, original title, name of substance word, subject heading word, keyword heading word, protocol supplementary concept word, rare disease supplementary concept word, unique identifier, synonyms]
2. Exp PRESCHOOL CHILDREN/
3. Exp INFANTS/
4. exp PAEDIATRICS/
5. 1 or 2 or 3 or 4
6. (HIV or HIV-1 or HIV-2 or human immunodeficiency virus or human immune deficiency virus or human immune-deficiency virus or HIV infect* or HIV-infected or living with HIV or HIV-positive or AIDS or Acquired Immunodeficiency Syndrome or Acquired Immune Deficiency Syndrome or HEU or HIV-exposed or HIV-exposed-uninfected or HIV-uninfected or HAART or antiretroviral* or anti-retroviral* or anti-HIV agents or HIV treatment or PMTCT or prevention of mother-to-child transmission).mp. [mp=title, abstract, original title, name of substance word, subject heading word, keyword heading word, protocol supplementary concept word, rare disease supplementary concept word, unique identifier, synonyms]
7. exp acquired immune deficiency syndrome/
8. exp human immunodeficiency viruses/ or exp hiv infections/ or exp human immunodeficiency virus 1/ or exp human immunodeficiency virus 2/
9. exp hiv fusion inhibitors/ or exp antiretroviral agents/ or exp hiv integrase inhibitors/ or exp hiv protease inhibitors/
10. exp reverse transcriptase inhibitors/ or exp non-nucleoside reverse transcriptase inhibitors/ or exp nucleoside reverse transcriptase inhibitors/ or exp nucleotide reverse transcriptase inhibitors/
11. exp antiviral agents/
12. 6 or 7 or 8 or 9 or 10 or 11
13. (Neurodevelop* or (develop* adj4 child) or neurocog* or cogniti* or communication or language or speech or verbal or motor or neuromotor or locomotor or neuroanatom* or (brain adj4 structure) or (brain adj4 microstructure) or (white adj4 matter) or head circumference or (developmental adj4 delay*) or (developmental adj4 disorder*) or (developmental adj4 disabilit*)).mp. [mp=title, abstract, original title, name of substance word, subject heading word, keyword heading word, protocol supplementary concept word, rare disease supplementary concept word, unique identifier, synonyms]
14. exp child development/
15. exp early childhood development/ or exp infant development/
16. exp pervasive child development disorders/

17. exp development/
18. exp cognitive development/ or exp psychomotor development/ or exp motor development/
19. exp speech development/
20. 13 or 14 or 15 or 16 or 17 or 18 or 19
21. 5 and 12 and 20

Africa-wide information

(infan* or newborn* or new-born* or perinat* or neonat* or baby or babies or toddler* or child* or pediatric* or paediatric*) AND (HIV or HIV-1 or HIV-2 or human immunodeficiency virus or human immune deficiency virus or human immune-deficiency virus or HIV infect* or HIV-infected or living with HIV or HIV-positive or AIDS or Acquired Immunodeficiency Syndrome or Acquired Immune Deficiency Syndrome or HEU or HIV-exposed or HIV-exposed-uninfected or HIV-uninfected or HAART or antiretroviral* or anti-retroviral* or anti-HIV agents or HIV treatment or PMTCT or prevention of mother-to-child transmission) AND (Neurodevelop* or (develop* N4 child) or neurocog* or cogniti* or communication or language or speech or verbal or motor or neuromotor or locomotor or neuroanatom* or (brain N4 structure) or (brain N4 microstructure) or (white N4 matter) or head circumference or (developmental N4 delay*) or (developmental N4 disorder*) or (developmental N4 disabilit*))

S3 Appendix: PECO inclusion and exclusion criteria

	Inclusion criteria	Exclusion criteria
**Population**	• Children aged 0 to 5 years<br>• Born after January 2000 (WHO first issued recommendations for antiretroviral drugs in 2000[1])	• Studies of children >5 years that did not include younger children*<br>• Children born before the year 2000
**Exposure**	• HIV exposure without HIV infection. HEU children were defined as children born to mothers with HIV infection with appropriate reporting confirming that children were not infected<br>• ART exposure was defined as exposure to any maternal antiretroviral drugs during pregnancy	• No documentation of maternal and child HIV infection status<br>• Children with HIV infection<br>• Studies where ART was noted to be unavailable at the time of the study<br>• Studies of HEU children where the primary scientific question related to other exposures and relevant data could not be extracted (e.g. nutritional deficiencies or other infections)
**Control**	• Children born to HIV-uninfected mothers (Aim 1) AND/OR<br>• HEU children exposed to different maternal ART regimens, classes or drugs or no treatment (Aim 2)	• Studies without either of these control groups
**Outcomes**	***Primary***:<br>• Cognitive development, receptive language, expressive language, fine motor, gross motor (as recommended for children 0-5 years corresponding to developmental areas in the ICD-10 and -11 Delayed Milestones definitions[2])<br>• Social-emotional and adaptive behaviour (defined as daily living skills that enable everyday function appropriate to the relevant age group[2]).<br>• Measured using validated instruments.<br>***Secondary***:<br>• Brain structure, as measured by neuroimaging<br>• Head circumference	• Other outcomes
**Study type**	• All primary data study designs<br>• Reviews and meta-analyses were hand-searched for additional primary studies<br>• Academic publications<br>• English or Spanish language	• Conference and poster abstracts<br>• Languages other than English and Spanish

*Studies were included if they had stratified analyses of the population of interest and the majority of children fulfilled the inclusion criteria. If the age range extended through 5 years, studies were included if the median age was <5 years.

S4 Appendix: Quality assessment tool
NIH Quality Assessment Tool for Observational Cohort and Cross-Sectional Studies criteria

1. Was the research question or objective in this paper clearly stated?
2. Was the study population clearly specified and defined?
3. Was the participation rate of eligible persons at least 50%?
4. Were all the subjects selected or recruited from the same or similar populations (including the same time period)? Were inclusion and exclusion criteria for being in the study prespecified and applied uniformly to all participants?
5. (5.a) Was a sample size justification, power description, or variance and effect estimates provided?
 (5.b) Was sample size ≥ 50 per group?*
6. For the analyses in this paper, were the exposure(s) of interest measured prior to the outcome(s) being measured?
7. Was the timeframe sufficient so that one could reasonably expect to see an association between exposure and outcome if it existed?
8. For exposures that can vary in amount or level, did the study examine different levels of the exposure as related to the outcome (e.g., categories of exposure, or exposure measured as continuous variable)? For the purposes of this review we assessed whether studies examined results by maternal CD4, viral load or ART.**
9. Were the exposure measures (independent variables) clearly defined, valid, reliable, and implemented consistently across all study participants? For the purposes of this review we assessed for valid HIV testing of mothers of HEU and HU children for ascertainment of exposure.***
10. Was the exposure(s) assessed more than once over time? For the purposes of this review we assessed valid HIV testing of HIV-exposed children and that children with HIV were excluded.***
11. Were the outcome measures (dependent variables) clearly defined, valid, reliable, and implemented consistently across all study participants?
12. Were the outcome assessors blinded to the exposure status of participants?
13. Was loss to follow-up after baseline 20% or less
14. Were key potential confounding variables measured and adjusted statistically for their impact on the relationship between exposure(s) and outcome(s)?

Risk of bias rating: Yes; No; CD, cannot determine; NA, not applicable; NR, not reported

Overall Study Quality rating: Good, Fair, Poor

* Question five was specifically adapted for this review per Carbia *et al*, 2018;
**Question eight was adapted for this review to assess if studies examined results by maternal CD4/viral load/ART to lend credibility to the hypothesis of causality between exposure and outcome;
***Given the importance of accurately assessing HIV exposure and avoidance of child HIV infection we adapted questions nine and ten to assess whether mothers of both HEU and HU children received valid HIV testing and that children who were HIV-exposed received valid HIV testing.

S5 Appendix: Characteristics of studies excluded following full-text review

No.	Year	Author	Title	Journal	Exclusion criteria
**1**	2009	Abubakar, A. *et al*	The role of weight for age and disease stage in poor psychomotor outcome of HIV-infected children in Kilifi, Kenya	*Dev Med Child Neurol*	No ART exposure – ART not routinely available at the time of the study
**2**	2013	Abubakar, A. *et al*	The performance of children prenatally exposed to HIV on the A-not-B task in Kilifi, Kenya: A preliminary study	*Int J Environ Res Public Health*	No ART exposure – ART not routinely available at the time of the study
**3**	2014	Abubakar, A.	Biomedical risk, psychosocial influences, and developmental outcomes: lessons from the pediatric HIV population in Africa	*New directions for child and adolescent development*	Review article; no new data.
**4**	2016	Ackermann, C. *et al*	Early antiretroviral therapy in HIV-infected children is associated with diffuse white matter structural abnormality and corpus callosum sparing	*American Journal of Neuroradiology*	No HEU group (parallel vaccine study included HEU and HU children combined in this paper); Age >5 years (control group mean 5.7 years)
**5**	2020	Ackermann, C *et al*	Diffusion tensor imaging point to ongoing functional impairment in HIV-infected children at age 5, undetectable using standard neurodevelopmental assessments	*AIDS Research and Therapy*	No distinct HEU group (control group of 11 from parallel vaccine study which included 9 HEU and 2 HU combined in this paper); Age >5 years included (control group mean 5.6 years)
**6**	2019	Ajayi, OR. *et al*	Association of anthropometric status and residential locality factors with cognitive scores of 4-6-year-old children in Kwazulu-Natal, South Africa	*South African Journal of Clinical Nutrition*	No HEU group; Age >5 years (4-6 years) and no stratification
**7**	2016	Alcock, KJ. *Et al*	The effect of prenatal HIV exposure on language functioning in Kenyan children: establishing an evaluative framework	*BMC Res Notes*	No ART exposure – ART not routinely available at the time of the study
**8**	2013	Andronikou, S.	Corpus callosum thickness on MRI as a surrogate marker of brain volume in children with HIV and its correlation with developmental scores and clinical parameters	*Pediatric Radiology*	No HEU group;<br>Conference presentation
**9**	2001	Bakaki, P. *et al*	Epidemiologic and clinical features of HIV-infected and HIV-uninfected Ugandan children younger than 18 months	*JAIDS*	No distinct HEU group - HEU and HU grouped together as 'HIV-uninfected'
**10**	2016	Bass, J.K. *et al*	Association of caregiver quality of care with neurocognitive outcomes in HIV-affected children aged 2-5 years in Uganda	*AIDS Care*	Cohort outcomes reported in Ruiseñor-Escudero *et al.*
**11**	2019	Blakstad, M.M. *et al*	Nutritional, socioeconomic, and delivery characteristics are associated with neurodevelopment in Tanzanian children	*Journal of Pediatrics*	HEU data reported in Manji KP *et al. (NCT00197730 trial)*
**12**	2001	Blanchette, N. *et al*	Cognitive and motor development in children with vertically transmitted HIV infection	*Brain and Cognition*	Cohort spanned years <2000
**13**	2016	Blokhuis, C. *et al*	The Eye as a Window to the Brain: Neuroretinal Thickness Is Associated With Microstructural White Matter Injury in HIV-Infected Children	*Investigative Ophthalmology & Visual Science*	No specified HEU group;<br>Age >5 years (mean 12.1 years)
**14**	2017	Boateng, G.O. *et al*	Early childhood learning activities buffer adverse effects of HIV exposure on infant cognitive development: A longitudinal study	*FASEB Journal*	No specified HEU group - 'HIV-exposed' group analysed which may include HIV-infected children; Poster presentation
**15**	2014	Boivin, M.J. *et al*	Bouts of malaria illness as mediated by anemia diminishes cognitive development in very young Ugandan children	*American Journal of Tropical Medicine and Hygiene*	Conference abstract (paper included in full reviews). No distinct HEU group – 'HIV-exposed' group analysed which may include HIV-infected children
**16**	2017	Boivin, M.J. *et al*	Effect of caregiver training on neurodevelopment of HIV-exposed uninfected children and caregiver mental health: a Ugandan cluster randomized controlled trial	*J Dev Behav Pediatr*	Intervention trial. ART exposure not mentioned
**17**	2018	Boivin, M.J. *et al*	Neuropsychological performance in African children with HIV enrolled in a multisite antiretroviral clinical trial	*AIDS*	Age >5 years (5-11 years)
**18**	2018	Boivin, M., *et al*	Developmental and cognitive effects of type of antepartum and postpartum ARV exposure for Ugandan and Malawian IMPACCT PROMISE HIV-exposed versus unexposed children at age 12, 24 and 48 months	*JIAS*	Conference presentation, paper included in full texts
**19**	2018	Boivin, M.J. *et al*	African multi-site 2-year study of neurocognition in HIV infected/affected children	*Topics in Antiviral Medicine (CROI 2018)*	Age >5 years (5-11 years)
**20**	2019	Boivin, M.J. *et al*	African multi-site 2-year neuropsychological study of school-age children perinatally infected, exposed, and unexposed to human immunodeficiency virus	*CID*	Age >5 years (5-11 years)
**21**	2020	Boivin, M.J. *et al*	Early Childhood Development Caregiver Training and Neurocognition of HIV-exposed Ugandan Siblings	*Journal of Developmental & Behavioral Pediatrics*	Age >5 years (5-12 years)
**22**	2008	Botha, J.A.E.	Motor development and growth status of 2 to 6 year old children infected with Human Immunodeficiency Virus [HIV]	*NA*	Thesis
**23**	2008	Botha, JAE & Pienaar AE.	The motor development of 2 to 6-year old children infected with HIV	*SA Journal for Research in Sport, Physical Education and Recreation*	No ART exposure – access to ART was limited at the time of the study
**24**	2011	Bowes, J. *et al*	Pervasive developmental disorder in antiretroviral- and HIV-exposed, uninfected children	*Canadian Journal of Infectious Diseases*	Cohort spanned years <2000 (1997-2010);<br>Conference presentation

				and Medical Microbiology	
**25**	2009	Briand, N. *et al*	No relation between in-utero exposure to HAART and intrauterine growth retardation	*AIDS*	Cohort spanned years <2000 (children born 1990 – 2006); although stratified by year only n=23 have head circumference measured in the >2000 group, <10% of cohort
**26**	2001	Bruck, I. *et al*	Developmental milestones of vertically HIV infected and seroreverters children: follow up of 83 children	*Arquivos de neuro-psiquiatria*	Cohort spanned years <2000 (children born 1995 – 2000)
**27**	2018	Budd, M.A. *et al*	Blood mitochondrial DNA content in HIV-exposed uninfected children with autism spectrum disorder	*Viruses*	Age > 5 years (2-16 years)
**28**	2003	Bulgheroni, S. *et al*	Longitudinal neuropsychological evaluation of neurologically asymptomatic HIV infected children	*Psicologia Clinica dello Sviluppo*	Age > 5 years included (4 – 15 years); Italian
**29**	2015	Buonomo, E., *et al*	Malnutrition decreases the odds of attaining motor milestones in HIV exposed children: results from a paediatric DREAM cohort	*Epidemiologia e prevenzione*	HEU group included HIV-infected child
**30**	2013	Cambrea, S.C. *et al*	Can HIV infection during pregnancy cause an intrauterine growth restriction?	*BMC Infectious Diseases*	No distinct HEU group - HEU and HIV-infected children grouped together; Conference presentation
**31**	2014	Cambrea, S.C. *et al*	Evaluation of anthropometric and virologic data in newborn from HIV positive mothers	*BMC Infectious Diseases*	No distinct HEU group - HEU and HIV-infected children grouped together; Conference presentation
**32**	2009	Carcellar, A. *et al*	Lack of effect on prematurity, birth weight, and infant growth from exposure to protease inhibitors in utero and after birth	*Pharmaco-therapy*	Cohort spanned years <2000 (1997-2005)
**33**	2020	Chandna, J. *et al*	Effects of improved complementary feeding and improved water, sanitation and hygiene on early child development among HIV-exposed children: substudy of a cluster randomized trial in rural Zimbabwe	*BMJ Global Health*	Main study results presented in Ntozini, R *et al,* included
**34**	2000	Chase, C. *et al*	Early cognitive and motor development among infants born to women infected with human immunodeficiency virus.	*Pediatrics*	Cohort spanned years <2000 (children born 1990 onwards)
**35**	2011	Chaworth-Musters, T. *et al*	Adverse health outcomes in HIV exposed uninfected children (HEU) in British Columbia - CIHR team grant in HIV therapy and aging (CARMA)	*Canadian Journal of Infectious Diseases and Medical Microbiology*	Age >5 years included (mean 5.4 years, range 0.6-19.6 years); Conference presentation
**36**	2018	Chernoff, M. *et al*	Assessing Psychiatric Symptoms in Youth Affected by HIV: Comparing a Brief Self-Administered Rating Scale with a Structured Diagnostic Interview	*Journal of Clinical Psychology in Medical Settings*	Age >5 years (range 6-18 years)
**37**	2018	Chernoff, M.C. *et al*	Validity of neuropsychological testing in young African children affected by HIV	*Journal of Pediatric Infectious Diseases*	Age >5 years (range 5-11 years)
**38**	2018	Chhaya, R. *et al*	The feasibility of an automated eye-tracking-modified Fagan test of memory for human faces in younger Ugandan HIV-exposed children	*Child Neuropsychology*	Feasibility study for automated eye-tracking. No mention of ART
**39**	2005	Chiriboga, C.A. *et al*	Incidence and prevalence of HIV encephalopathy in children with HIV infection receiving highly active anti-retroviral therapy (HAART)	*Journal of Pediatrics*	No HEU group; Cohort spanned years <2000 (followed up from 1988 onwards)
**40**	2018	Chingono, R. *et al*	Evaluating the effectiveness of a multi-component intervention on early childhood development in paediatric HIV care and treatment programmes: a randomized controlled trial	*BMC Pediatrics*	Trial protocol, no results
**41**	2019	Christodoulou, J. *et al*	Perinatal maternal depression in rural South Africa: Child outcomes over the first two years	*Journal of Affective Disorders*	No HEU group or stratification by HIV status
**42**	2019	Cockcroft, K. and Milligan, R.	Working memory structure in atypical development: HIV-infected and HIV-exposed, uninfected school beginners	*Developmental Neuropsychology*	Age >5 years (HEU mean age 7.36 years, SD 0.88)
**43**	2017	Coelho, A.V. *et al*	Antiretroviral Treatment in HIV-1-Positive Mothers: Neurological Implications in Virus-Free Children	*International Journal of Molecular Sciences*	Review
**44**	2015	Conserve, D.G., *et al*	Maternal HIV illness and its impact on children well-being and development in Haiti	*J Child Fam Stud*	No neurodevelopment outcomes; focus on caregiver not children; no distinct HEU group
**45**	2018	Dalili, D. *et al*	Growth and development status in the first two years of uninfected children born from HIV positive mothers	*Acta Medica Iranica*	No HU control group or statistical comparison with normative data
**46**	2016	Das, P.K. *et al*	Abundance of psychiatric morbidity in perinatally HIV infected children and adolescents with comparison to their HIV negative sibling	*Neurology Psychiatry and Brain Research*	Age >5 years included (stratification at 6 years)
**47**	2016	Datong, P *et al*	Breast-fed HIV-1 exposed infants play catch up	*BMC Infectious Diseases Conference*	Conference abstract; No distinct HEU group – no statement HIV exposed children are all uninfected
**48**	2000	Davis-McFarland, E.	Language and oral-motor development and disorders in infants and young toddlers with human immunodeficiency virus	*Seminars in speech and language*	Review of HIV-infected children
**49**	2018	Debeaudrap, P. *et al*	Neurodevelopmental outcomes in HIV-infected and uninfected African children	*AIDS*	Age >5 years (4-9 years, mean HEU 6.2 years, HU 6.2 years)
**50**	2003	Del Pilar Kufa, M. *et al*	Neurodevelopment in HIV-exposed children	*Interdisciplinaria Revista de*	No distinct HEU group – infants born to HIV-infected women analysed which may include HIV-infected children

				Psicologia y Ciencias Afines	
**51**	2016	Desmonde, S.T. *et al*	Health and survival of HIV perinatally exposed but uninfected children born to HIV-infected mothers	*Current opinion in HIV and AIDS*	Review
**52**	2019	Donald, K.A. *et al*	Risk and protective factors for child development: An observational South African birth cohort	*PLOS Medicine*	HEU outcomes reported in *Wedderburn, C.J. et al*
**53**	2019	Ekali, G.L. *et al*	Effect of in utero exposure to HIV and antiretroviral drugs on growth in HIV-exposed uninfected children: a systematic review and meta-analysis protocol	*BMJ Open*	Review; protocol without results
**54**	2016	Evans, C.V. *et al*	Head circumferences of children born to HIV-infected and HIV-uninfected mothers in Zimbabwe during the preantiretroviral therapy era	*AIDS*	Cohort spanned years <2000 (1997-2000)
**55**	2016	Ezeamama, A.E. *et al*	Perinatal HIV Status and Executive Function During School-Age and Adolescence: A Comparative Study of Long-Term Cognitive Capacity Among Children From a High HIV Prevalence Setting	*Medicine*	Age >5 years (cognitive assessment between 6-18 years, school age)
**56**	2019	Ezeamama, A.E. *et al*	Serum vitamin D is differentially associated with socioemotional adjustment in early school-aged Ugandan children according to perinatal HIV status and in utero or peripartum antiretroviral exposure history	*American Journal of Tropical Medicine and Hygiene*	Age >5 years (6-10 years, mean HEU 7.5 years, HUU 7.6 years); conference presentation (full paper checked: Yakah, W. *et al* Nutrients 2019;11(7):1570)
**57**	2020	Familiar, I. *et al*	Association between caregiver depression symptoms and child executive functioning. Results from an observational study carried out in four sub-Saharan countries	*AIDS Care*	Age >5 years (5-11 years; HEU mean 7.3 years, HUU 7.3 years)
**58**	2014	Fasunla, A.J.O.G. *et al*	Comparison of hearing status of HIV-exposed and-unexposed newborns and immunovirologic correlates in HIV-exposed newborns	*Otolaryngology - Head and Neck Surgery (United States)*	Conference abstract; No distinct HEU group - 'HIV-exposed' group analysed which may include HIV-infected children
**59**	2014	Fasunla A.J. *et al*	Comparison of auditory brainstem response in HIV-1 exposed and unexposed newborns and their correlation with the maternal viral load and CD4 cell counts	*AIDS*	Paper from above; HEU group not clearly defined; Outcome not included.
**60**	2009	Ferguson, G. *et al*	The prevalence of motor delay among HIV infected children living in Cape Town, South Africa	*International Journal of Rehabilitation Research*	No specified HEU group – reference sample unknown HIV-status
**61**	2011	Ferguson, G. *et al*	The motor development of orphaned children with and without HIV: Pilot exploration of foster care and residential placement	*Physiotherapy (United Kingdom)*	Conference abstract; No HEU group
**62**	2013	Ferguson, K.T. *et al*	Cognitive, motor, and behavioral development of orphans of HIV/AIDS in institutional contexts	*Neuropsychology of children in Africa: Perspectives on risk and resilience*	No distinct HEU group (20% of children had been diagnosed with HIV/AIDS)
**63**	2009	Fernández Ibieta, M. *et al*	Growth of uninfected infants exposed to antiretrovirals born to HIV-infected women	*Anales de Pediatria*	No control group
**64**	2000	Fishkin, P.E. *et al*	Brief report: relationship between HIV infection and WPPSI-R performance in preschool-age children	*Journal of Pediatric Psychology*	No HEU group;<br>Cohort spanned years <2000 (1999+)
**65**	2018	Fouché, C. *et al*	Anthropometric parameters of HIV-infected and HIV-uninfected mothers and their premature infants	*Journal of Tropical Pediatrics*	No distinct HEU group - 'HIV-infected mothers and their premature infants analysed which may include HIV-infected children
**66**	2019	Gaulee Pokhrel, K. V. D. *et al*	Influence of stigma and discrimination on psychosocial health in children affected by AIDS in Nepal: A cross-sectional study	*HIV Medicine*	No distinct HEU group; Age > 5 years (2-14 years); Conference abstract
**67**	2020	Ge, X.M. *et al*	Influence on physical development of children aged 18 months from HIV-positive mothers for prevention mother to child transmission of HIV	*Zhonghua liu xing bing xue za zhi*	Anthropometry measures grouped, no head circumference. Language of full-text: Chinese
**68**	2017	Gonzalez, R. *et al*	Effects of HIV infection on maternal and neonatal health in southern Mozambique: a prospective cohort study after a decade of antiretroviral drugs roll out	*PLoS ONE*	No distinct HEU group - infants born to HIV-infected women which may include HIV-infected children
**69**	2012	Gompels, U.A *et al*	Human Cytomegalovirus Infant Infection Adversely Affects Growth and Development in Maternally HIV-Exposed and Unexposed Infants in Zambia	*CID*	No distinct HEU group - separated by maternal HIV status but not combined with child status so HIV-infected children included with HEU group
**70**	2011	Griner, R. *et al*	In utero and postnatal exposure to antiretrovirals among HIV-exposed but uninfected children in the United States	*AIDS Patient Care STDS*	Cohort spanned years <2000 (1995 to 2009); No neurodevelopmental outcomes
**71**	2008	Grosch-Woerner, I. *et al*	Increased rate of prematurity associated with antenatal antiretroviral therapy in a German/Austrian cohort of HIV-1-infected women	HIV Medicine	Cohort spanned years <2000 (1995-2001)
**72**	2005	Hankin, C. *et al*	Does exposure to antiretroviral therapy affect growth in the first 18 months of life in uninfected children born to HIV-infected women?	*JAIDS*	Cohort spanned years <2000 (1985+)
**73**	2018	Heffron, R. *et al*	Pregnancy outcomes and infant growth among babies with in-utero exposure to tenofovir-based preexposure prophylaxis for HIV prevention	*AIDS*	No HEU children
**74**	2016	Hermetet-Lindsay, K.D.	Contributions of disease severity, psychosocial factors, and cognition to behavioral functioning in youth perinatally exposed to HIV	*Dissertation Abstracts*	Age includes >5 years (school age: mean 10.9 years); Dissertation

**75**	2013	Herrero, D. *et al*	Motor development of infants exposed to maternal human immunodeficiency virus (HIV) but not infected	*International Archives of Medicine*	No HU control group or statistical comparison with normative data.
**76**	2015	Himes, S.K. *et al*	Meconium atazanavir concentrations and early language outcomes in HIV-exposed, uninfected infants with prenatal atazanavir exposure	*JAIDS*	No control group. Focus on meconium concentrations
**77**	2008	Hochhauser, C.J. *et al*	The impact of environmental risk factors on HIV-associated cognitive decline in children	*AIDS Care*	No HEU group; Cohort spanned years <2000 (1993-onwards); Age >5 years (1-13 years)
**78**	2001	Holditch-Davis, D. *et l*	Parental caregiving and developmental outcomes of infants of mothers with HIV	*Nursing research*	Cohort spanned years <2000 (children born before year 2000).No distinct HEU group
**79**	2011	Hutchings, J.	Developmental delay in HIV-exposed infants attending Newlands Clinic in Harare, Zimbabwe	*NA*	Dissertation
**80**	2014	Hutchings, J. & Potterton, J.	Developmental delay in HIV-exposed infants in Harare, Zimbabwe	*Vulnerable children and youth studies*	No HU control group or statistical comparison with normative data.
**81**	2019	Iloghalu, E.I. *et al*	Effect of maternal HIV infection on treatment with HAART on neonatal birth weight and other anthropometry: A cohort study of HIV sero-positive women in Enugu, South-East Nigeria	*Journal of Clinical and Diagnostic Research*	No distinct HEU group - infants born to HIV sero-positive women which may include HIV-infected children
**82**	2017	Iloh, K.K. *et al*	Neurocognitive function of school-aged HIV-infected children in Enugu, Nigeria	*Journal of Tropical Pediatrics*	No specified HEU group;<br>Age>5 years (6-15 years)
**83**	2018	Jacobson, D. *et al*	Alcohol use among HIV-infected pregnant women and child outcomes in the Pediatric HIV AIDS Cohort study (PHACS)	*Alcoholism: Clinical and Experimental Research*	Conference presentation; substance use confounder; age range for neurodevelopment evaluations not clear (children followed up until age 18)
**84**	2017	Jankiewicz, M. *et al*	White matter abnormalities in children with HIV infection and exposure	*Frontiers in Neuroanatomy*	Age (follow up >5 years; mean age 7.3 years)
**85**	2020	Jantarabenjakul W. *et al*	Behavioral problems in perinatally HIV-infected young children with early antiretroviral therapy and HIV-exposed uninfected young children: prevalence and associated factors	*AIDS Care*	Neurodevelopmental outcomes reported in other paper (*Jantarabenjakul W. et al., 2019)*
**86**	2019	Jantarabenjakul W. *et al*	Low risk of neurodevelopmental impairment among perinatally acquired HIV-infected preschool children who received early antiretroviral treatment in Thailand	*JIAS*	No HU control group or statistical comparison with normative data
**87**	2015	Jao, J. *et al*	Growth patterns in the first year of life differ in infants born to perinatally vs. non-perinatally HIV-infected women	*AIDS*	No neurodevelopmental outcomes or OFC
**88**	2020	Jao, J. *et al*	Neurodevelopment of HIV-exposed uninfected infants born to women with perinatally acquired HIV in the United States	*J Acquir Immune Defic Syndr*	Comparison of perinatally versus non-perinatally acquired HIV in mothers. SMARTT cohort and neurodevelopmental outcomes reported in included papers
**89**	2011	Jelsma, J. *et al*	The motor development of orphaned children with and without HIV: Pilot exploration of foster care and residential placement	*BMC Pediatrics*	No distinct HEU group (children infected with HIV compared with those without HIV residing in institutions or with foster parents, maternal HIV status not documented)
**90**	2016	Kaaya, S. *et al*	Association of maternal depression and infant nutritional status among women living with HIV in Tanzania	*Maternal & child nutrition*	No neurodevelopmental outcomes;<br>20% infants of unknown HIV status; cohort spanned years <2000 (1995 – 1997)
**91**	2016	Kakkar, F.W. *et al*	Safety of combination antiretroviral prophylaxis in high-risk HIV-exposed newborns: A retrospective review of the Canadian experience	*JIAS*	Cohort spanned years <2000 (1997-2013); No distinct HEU group - 'high-risk HIV-exposed' group analysed which may include HIV-infected children
**92**	2012	Kapetanovic, S. *et al*	T-cell activation and neurodevelopmental outcomes in perinatally HIV-infected children	*AIDS*	No HEU group
**93**	2004	Keller, M.A. *et al*	Altered neurometabolite development in HIV-infected children: Correlation with neuropsychological tests	*Neurology*	No specified HEU group;<br>Age >5 years (range 6-16 years)
**94**	2014	Kerr, S.J. *et al*	Neurodevelopmental outcomes in HIV-exposed-uninfected children versus those not exposed to HIV	*AIDS Care*	Age includes >5 years and no subanalysis (1-12 years, mean 7.6 years
**95**	2000	Knight, W.G. *et al*	Brief report: Effects of pediatric HIV infection on mental and psychomotor development	*Journal of Pediatric Psychology*	Cohort spanned years <2000
**96**	2018	Knox, J. *et al*	Screening for developmental disabilities in HIV positive and HIV negative children in South Africa: Results from the Asenze Study	*PLoS One*	No distinct HEU group - separated by child HIV status but not the combined HEU v. HUU groupings – 'HIV-negative group contains HEU and HU children
**97**	2004	Kullgren, K.A. *et al*	Prediction of cognitive, adaptive, and behavioral functioning in preschool and school-age children with HIV	*Children's Health Care*	No HEU group; Age > 5 years (3 to 16 years and no stratification)
**98**	2014	Kuona, P. *et al*	Growth and development of the HIV exposed uninfected children below 5 years in developing countries: focus on nutritional challenges, mortality and neurocognitive function. (Special issue on malnutrition.)	*Food and Nutrition Sciences*	Review
**99**	2000	Layton, T. *et al*	Language development and assessment in children with human immunodeficiency virus: 3 to 6 years	*Seminars in speech and language*	Review
**100**	2012	Le Doare, K. *et al*	Neurodevelopment in children born to HIV-infected mothers by infection and treatment status	*Pediatrics*	Review
**101**	2018	Le Roux, S.M. *et al*	HIV viraemia in pregnancy and neurodevelopment of HIV-exposed uninfected children	*Topics in Antiviral Medicine*	Conference presentation

**102**	2018	Le Roux, S.M. *et al*	HIV viremia during pregnancy and neurodevelopment of HIV-exposed uninfected children in the context of universal antiretroviral therapy and breastfeeding	*HIV Reports*	Neurodevelopmental outcomes reported in separate paper Le Roux et al 2018, included in analysis
**103**	2019	Le Roux, K. *et al*	A longitudinal cohort study of rural adolescent vs adult South African mothers and their children from birth to 24 months	*BMC Pregnancy and Childbirth*	No HEU group - only HIV status of mother described
**104**	2009	Li, Y. *et al*	A study on clinical characteristics, growth development, and intelligence of child patients with Human Immunodeficiency Virus type	*Chinese Journal of Clinical Psychology*	Language of full-text: Chinese; No distinct HEU group - 'HIV-infected versus normal children'
**105**	2016	Li, Y.X. *et al*	Physical development of HIV-exposed infants in Kunming city: a cohort study	*Maternal and Child Health Care of China*	Language of full-text: Chinese; No developmental outcomes
**106**	2009	Lin, XY. *et al*	Physical and psychological health among children affected by HIV/AIDS: Difference in groups and caring arrangements	*Chinese Journal of Clinical Psychology*	Language of full-text: Chinese; No distinct HEU group - 'Participants were double AIDS orphans, single AIDS orphans and affected children who were taken care of by family and orphanage'
**107**	2007	Lindsey, J.C. *et al*	Neurodevelopmental functioning in HIV-infected infants and young children before and after the introduction of protease inhibitor-based highly active antiretroviral therapy	*Pediatrics*	Cohort spanned years <2000 (1993-onwards; cohort demographics grouped by year of birth but not development)
**108**	2015	Linn, K. *et al*	HIV-Related Cognitive Impairment of Orphans in Myanmar with Vertically Transmitted HIV Taking Antiretroviral Therapy	*Pediatric Neurology*	No distinct HEU group - 'HIV-infected versus HIV-negative children in orphanages' No further information on maternal HIV status of HIV-negative children
**109**	2016	Liotta, G. *et al*	Growth indices in breastfed infants pre and postnatally exposed to tenofovir compared with tenofovir-unexposed infants	*AIDS*	No neurodevelopmental outcomes
**110**	2002	Lipman, T.H. *et al*	Assessment of growth and immunologic function in HIV-infected and exposed children	*Journal of the Association of Nurses in AIDS Care*	No neurodevelopmental outcomes; Includes children >5 years (0-14 years)
**111**	2016	Louw, K.A. *et al*	Correlates of emotional and behavioural problems in children with perinatally acquired HIV in Cape Town, South Africa	*AIDS Care*	No HEU group; Age includes >5 years (6-16 years)
**112**	2001	Macmillan, C. *et al*	Head growth and neurodevelopment of infants born to HIV-1-infected drug-using women	*Neurology*	Cohort spanned years <2000); Focused on opiates and cocaine
**113**	2011	Malee, K.M. *et al*	Mental health functioning among children and adolescents with perinatal HIV infection and perinatal HIV exposure	*AIDS Care*	Age >5 years (7-16yrs)
**114**	2016	Malee, K.M. *et al*	Brain and Cognitive Development Among U.S. Youth With Perinatally Acquired Human Immunodeficiency Virus Infection	*Journal of the Pediatric Infectious Diseases Society*	Review
**115**	2011	Manfredi, A.K. *et al*	Newborn hearing screening in infants born to HIV-seropositive mothers	*J Soc Bras Fonoaudiol*	No distinct HEU group - 'HIV-exposed' group analysed which may include HIV-infected children
**116**	2014	Manji, K.P. *et al*	Effect of multivitamin supplementation on the neurodevelopment of HIV-exposed Tanzanian infants: a randomized, double-blind, placebo-controlled clinical trial	*J. Tropical Pediatrics*	Focus on multivitamin supplementation
**117**	2012	Manno, D. *et al*	Rich micronutrient fortification of locally produced infant food does not improve mental and motor development of Zambian infants: a randomised controlled trial	*British Journal of Nutrition*	No distinct HEU group - separated by child HIV status and maternal HIV status but not the combined HEU v. HU groupings
**118**	2018	Marques, K.C. *et al*	Motor coordination of children and adolescents with human immunodeficiency virus	*Ciência & Saúde*	Language: Portuguese; Age > 5 years
**119**	2012	Martinez, P.C. *et al*	Intellectual quotient score comparison between HIV-infected and HIV exposed children at the Peruvian national institute of child health, Lima Peru	*Retrovirology (Conference)*	Age >5 years (3 - 7 years and no stratification); Conference presentation
**120**	2012	McDonald, C. *et al*	Morbidity and undernutrition are associated with impaired neurodevelopment among HIV-exposed infants in Tanzania	*FASEB Journal. Conference: Experimental Biology*	Conference abstract of 2013 McDonald *et al* paper; Insufficient information for HEU analysis
**121**	2013	McDonald, C. *et al*	Effect of multiple micronutrient supplementation on the neurodevelopment of HIV-exposed Tanzanian infants	*FASEB Journal. Conference: Experimental Biology*	Conference abstract of 2014 Manji *et al* paper.
**122**	2013	McDonald, C. *et al*	Stunting and wasting are associated with poorer psychomotor and mental development in HIV-exposed Tanzanian infants	*Journal of Nutrition*	Cohort spanned years <2000 (pregnant women recruited 1995 to 1997)
**123**	2006	McGrath, N *et al*	Effect of maternal multivitamin supplementation on the mental and psychomotor development of children who are born to HIV-1-infected mothers in Tanzania	*Pediatrics*	Cohort spanned years <2000 (pregnant women recruited 1995 to 1997); Not analysed by HEU status
**124**	2006	McGrath, N *et al*	The timing of mother-to-child transmission of human immunodeficiency virus infection and the neurodevelopment of children in Tanzania	*PIDJ*	Cohort spanned years <2000 (pregnant women recruited 1995 to 1997)
**125**	2018	McHenry, M.S. *et al*	Neurodevelopment in Young Children Born to HIV-Infected Mothers: A Meta-analysis	*Pediatrics*	Review
**126**	2019	McHenry, M.S. *et al*	In utero exposure to HIV and/or antiretroviral therapy: a systematic review of preclinical and clinical evidence of cognitive outcomes	*JIAS*	Review

**127**	2019	McHenry, M.S. *et al*	Interventions for developmental delays in children born to HIV-infected mothers: a systematic review	*AIDS Care*	Review
**128**	2018	McHenry, M.S. *et al*	Early childhood development in children born to HIV-infected mothers: perspectives from Kenyan clinical providers and caregivers	*Glob Pediatr Health*	No neurodevelopmental outcomes measured
**129**	2018	Mebrahtu, H. *et al*	Postpartum maternal mental health is associated with cognitive development of HIV-exposed infants in Zimbabwe: a cross-sectional study	*AIDS Care*	No ART exposure documented. Children with HIV included in the analysis
**130**	2019	Mebrahtu, H. *et al*	Effects of parenting classes and economic strengthening for caregivers on the cognition of HIV-exposed infants: a pragmatic cluster randomized controlled trial in rural Zimbabwe	*BMJ Global Health*	Developmental outcomes not assessed by HEU status; same trial [CHIDO] as included Mebrahtu *et al* 2018 paper
**131**	2019	Mebrahtu, H. *et al*	The impact of common mental disorders among caregivers living with HIV on child cognitive development in Zimbabwe	*AIDS Care*	Developmental outcomes not assessed by HEU status; same trial [CHIDO] as included Mebrahtu *et al* 2018 paper
**132**	2018	Mellins, C.A. *et al*	Screening for mental health among young South African children: the use of the Strengths and Difficulties Questionnaire (SDQ)	*Global Social Welfare*	Developmental outcomes not assessed by HEU status
**133**	2017	Milligan, R. *et al*	Working memory profiles in HIV-exposed, uninfected and HIV-infected children: A comparison with neurotypical controls	*Frontiers in Human Neuroscience*	Age >5years (mean age HEU group 88.28 months)
**134**	2019	Moraka, N.O. *et al*	Child HIV exposure and CMV seroprevalence in Botswana: No associations with 24-month growth and neurodevelopment	*Open Forum Infectious Diseases*	Analysis by CMV status and not HEU status; Children from the Tshipidi study included in Chaudhury *et al* paper
**135**	2013	Muhangi, L. *et al*	Maternal HIV infection and other factors associated with growth outcomes of HIV-uninfected infants in Entebbe, Uganda	*Public Health Nutrition*	No neurodevelopmental outcomes
**136**	2019	Mukherjee, S.B. *et al*	Development, cognition, adaptive function and maladaptive behavior in HIV-infected and HIV-exposed uninfected children aged 2-9 years	*Indian Pediatrics*	Age includes >5 years (2-9 years; HEU group mean 6.1 years) and no stratification
**137**	2017	Munoz, M *et al*	Community-Based Needs Assessment of Neurodevelopment, Caregiver, and Home Environment Factors in Young Children Affected by HIV in Lima, Peru	*Journal of the International Association of Providers of AIDS Care*	7 HEU children only and no HEU group analysis (only child and maternal status analysed separately)
**138**	2018	Murthy, V. *et al*	A study of neuropsychological profile of human immunodeficiency virus-positive children and adolescents on antiretroviral therapy	*Indian Journal of Psychiatry*	No specified HEU group; Age >5 years (8-15 years)
**139**	2017	Nachega, J.B. *et al*	Safety of tenofovir disoproxil fumarate-based antiretroviral therapy regimens in pregnancy for HIV-infected women and their infants: A systematic review and meta-analysis	*JAIDS*	Review
**140**	2014	Ngoma, M. *et al*	No evidence of neurodevelopmental delay in HEU infants exposed to cART in utero and breastfeeding	*Topics in Antiviral Medicine*	Conference presentation; duplicate of Ngoma *et al* 2014 paper included in analysis
**141**	2014	Ngoma, M. *et al*	Zambian HIV-Exposed Uninfected (HEU) infants exposed to HAART during pregnancy and one year of breastfeeding show no evidence of neurodevelopmental delay compared to HIV-Unexposed Uninfected (HUU) infants from the same community	*Canadian Journal of Infectious Diseases and Medical Microbiology*	Conference presentation; duplicate of Ngoma *et al* 2014 paper included in analysis
**142**	2012	Nielsen-Saines, K. *et al*	Infant outcomes after maternal antiretroviral exposure in resource-limited settings	*Pediatrics*	Included children with HIV in the analysis
**143**	2019	Obiagwu, P.N	Gross motor developmental delay in human immunodeficiency virus-infected children under 2 years of age	*Annals of African Medicine*	No HEU group (children were tested for HIV but mothers were only tested if children were HIV-infected)
**144**	2019	Onyango-Makumbi, C.	Extended prophylaxis with nevirapine does not affect growth in HIV-exposed infants	*JAIDS*	No maternal ART exposure comparison group (compared postnatal prophylaxis regimen)
**145**	2019	Pamplona, M.C.C.A. *et al*	Influence of exposure and vertical transmission of HIV-1 on the neuropsychomotor development in children'	*Revista da Sociedade Brasileira de Medicina Tropical*	No distinct HEU group (children born to mothers with HIV-1 infection compared to those born to mothers without HIV-1 infection, at least one HIV-infected child included in exposed group)
**146**	2010	Patel, D. *et al*	Breastfeeding, HIV status and weights in South African children: a comparison of HIV-exposed and unexposed children	*AIDS*	No neurodevelopmental outcomes
**147**	2005	Paul, M.E. *et al*	Morbidity and mortality during the first two years of life among uninfected children born to human immunodeficiency virus type 1-infected women: the women and infants transmission study	*PIDJ*	Cohort spanned years <2000 (enrolled by 1999)
**148**	2018	Paul, R. *et al*	Cognition, emotional health, and immunological markers in children with long-term nonprogressive HIV	*JAIDS*	Age >5 years (HEU median 6.8 [5.0-9.8]; HUU 7.4 [5.3 – 9.8])
**149**	2015	Perez, E.M. *et al*	Massage therapy improves the development of HIV-exposed infants living in a low socio-economic, peri-urban community of South Africa	*Infant Behavior & Development*	No HU control group or statistical comparison with normative data. Intervention study

**150**	2017	Pham, A. *et al*	Prenatal anti-retroviral exposure: An exploratory study of neurodevelopmental outcome in non-infected 5-years-old children	*European Journal of Paediatric Neurology*	Conference presentation, insufficient information
**151**	2018	Phillips, N.J. *et al*	HIV-associated cognitive disorders in perinatally infected children and adolescents: a novel composite cognitive domains score	*AIDS Care*	No specified HEU group;<br>Age >5 year (9-12 years)
**152**	2016	Pierre, R.B. *et al*	Infectious disease morbidity and growth among young HIV-exposed uninfected children in Jamaica	*Pan American Journal of Public Health*	No neurodevelopmental outcomes
**153**	2018	Piske, M. *et al*	Developmental outcomes and ARV exposure in HIV-exposed uninfected children	*Topics in Antiviral Medicine*	Cohort spanned years <2000 (1990-2012); Age included >5 years. Conference abstract
**154**	2018	Piske, M. *et al*	Neurodevelopmental outcomes and in-utero antiretroviral exposure in HIV-exposed uninfected children	*AIDS*	Cohort spanned years <2000 (1990-2012); Model 3 presented children born between 1 April 2000 to 31 December 2012 but age included >5 years
**155**	2007	Potterton, J.L.	A longitudinal study of neurodevelopmental delay in HIV infected children	*NA*	Thesis; No distinct HEU group
**156**	2001	Potterton, J.L. & Eales, C.J.	Prevalence of developmental delay in infants who are HIV positive	*South African Journal of Physiotherapy*	No distinct HEU group
**157**	2016	Powis, K.M. *et al*	In-utero triple antiretroviral exposure associated with decreased growth among HIV-exposed uninfected infants in Botswana	*AIDS*	No neurodevelopmental outcomes
**158**	2011	Powis, K.M. *et al*	Effects of in utero antiretroviral exposure on longitudinal growth of HIV-exposed uninfected infants in Botswana	*JAIDS*	No neurodevelopmental outcomes
**159**	2018	Purswani, M. *et al*	Birth prevalence of congenital cytomegalovirus infection and language, hearing and developmental outcomes in a cohort of HIV-exposed, uninfected preschool children	*Open Forum Infectious Diseases*	Conference presentation of included paper *Purswani et al.*
**160**	2019	Purswani, M. *et al*	Birth prevalence of congenital cytomegalovirus infection in HIV-exposed uninfected children in the era of combination antiretroviral therapy	*JPEDS*	Focus on congenital cytomegalovirus
**161**	2010	Puthanakit, T. *et al*	Poor cognitive functioning of school-aged children in Thailand with perinatally acquired HIV infection taking antiretroviral therapy	*AIDS Patient Care and STDs*	Age > 5 years; (6-12 years)
**162**	2013	Puthanakit, T. *et al*	Cognitive function and neurodevelopmental outcomes in HIV-infected children older than 1 year of age randomized to early versus deferred antiretroviral therapy: The PREDICT neurodevelopmental study	*Paediatr Infect Dis J*	Age > 5 years and no stratification or sub-analysis (median 7 years). Of the 155 HEU children, 40 <5 years; of the 164 HU children, 38 <5 years.
**163**	2013	Ransom, C.E. *et al*	Infant growth outcomes after maternal tenofovir disoproxil fumarate use during pregnancy	*JAIDS*	No neurodevelopmental outcomes
**164**	2016	Redmond, S.M. *et al*	Longitudinal Evaluation of Language Impairment in Youth With Perinatally Acquired Human Immunodeficiency Virus (HIV) and Youth With Perinatal HIV Exposure	*Journal of the Pediatric Infectious Diseases Society*	Age >5 years (7 - 16 years)
**165**	2017	Reliquet, V. *et al*	Developmental delay and behavioral disorders in 59 HIV-exposed uninfected infants	*Translational Pediatrics*	Age includes >5 years and no sub-analysis (1.8-11.7 years follow up)
**166**	2016	Rice, M.L. *et al*	ARV risk for speech and language impairments in HEU children at 3 and 5 years	*Topics in Antiviral Medicine*	Conference presentation of included paper *Rice, M.L. et al 2018*
**167**	2005	Rocha, C. *et al*	Neurological findings in a group of children and adolescents exposed and infected by HIV-1.	*Arquivos de Neuro-Psiquiatria*	Language: Portuguese
**168**	2018	Rodriguez, V.J. *et al*	Pre- and postnatal exposure to intimate partner violence among South African HIV-infected mothers and infant developmental functioning at 12 months of age	*Archives of Women's Mental Health*	No distinct HEU group - 'HIV-exposed' group analysed which included 4 HIV-infected children'
**169**	2018	Rodriguez, V.J. *et al*	Infant development and pre- and post-partum depression in rural South African HIV-infected women	*AIDS & Behavior*	No distinct HEU group - 'HIV-exposed' group analysed which included 4 HIV-infected children'
**170**	2017	Rosala-Hallas, A. *et al*	Growth of HIV-exposed uninfected, compared with HIV-unexposed, Zambian children: a longitudinal analysis from infancy to school age	*BMC Pediatrics*	No neurodevelopmental outcomes
**171**	2019	Rotheram-Borus, M. J. *et al*	Maternal HIV does not affect resiliency among uninfected/HIV exposed South African children from birth to 5 years	*AIDS*	No disaggregation of results by HEU group – neurodevelopmental results presented for resilient group versus non-resilient group
**172**	2018	Rotheram-Fuller, E.J.	Maternal patterns of antenatal and postnatal depressed mood and the impact on child health at 3 years postpartum	*Journal of Consulting and Clinical Psychology*	No distinct HEU group - HIV-infected children excluded but analysis combined exposed and unexposed uninfected children
**173**	2019	Ruiseñor-Escudero, H. *et al*	Building capacity in neurodevelopment assessment of children in sub-Saharan Africa: A quality assurance model to implement standardized neurodevelopment testing	*Child Neuropsychol*	No results by HEU exposure presented; Age >5 years (mean across 6 sites 7.2 years)
**174**	2019	Ruiseñor-Escudero, H. *et al*	Neurodevelopmental outcomes in preschool children living with HIV-1 subtypes A and D in Uganda	*HIV reports*	No HU control group or statistical comparison with normative data
**175**	2016	Ruskowski, A. *et al*	The role of maternal vitamin D and iron status on developmental outcomes and head circumference in HIV-exposed uninfected infants	*Journal of Pediatric Gastroenterology and Nutrition*	Conference presentation; insufficient information

**176**	2015	Sa, C.S.C. *et al*	Motor and cognitive developmental of children exposed and no exposed to HIV	*Physiotherapy (United Kingdom)*	Conference abstract; included paper *da Silva et al, 2015*
**177**	2012	Salihu, H.M. *et al*	Maternal HIV/AIDS status and neurological outcomes in neonates: A population-based study	*Maternal and Child Health Journal*	Cohort spanned years <2000 (1998-2007)
**178**	2005	Sanmaneechai, O. *et al*	Growth, developmental, and behavioral outcomes of HIV-affected preschool children in Thailand	*Journal of the Medical Association of Thailand*	Population spanned years <2000 (children born 1998-2000)
**179**	2017	Shariat, M. *et al*	Growth and neurodevelopmental status in HIV infected children	*Iranian Journal of Pediatrics*	No HEU group
**180**	2007	Shaw, R.R.	The relationship between pediatric HIV infection, CD4 percentage, HAART, and WISC-III performance	*Dissertation Abstracts International Section A:*	Dissertation abstract; Insufficient information
**181**	2010	Shead, G.M. *et al*	Neurodevelopment and growth of institutionalized children with vertically transmitted human immunodeficiency virus	*Vulnerable Children and Youth Studies*	No HEU group (HIV-uninfected children, but no indication of maternal HIV status)
**182**	2014	Sherr, L. *et al*	A systematic review of psychological functioning of children exposed to HIV: using evidence to plan for tomorrow's HIV needs	*AIDS and behavior*	Review
**183**	2018	Sherr, L. *et al*	Cognitive and physical development in HIV-positive children in South Africa and Malawi: A community-based follow-up comparison study	*Child: Care, Health and Development*	Age includes >5 years (4-13 years) and no sub-analysis
**184**	2012	Shet, A. *et al*	Cognitive, neurological and adaptive behaviour functioning among children with perinatally-acquired HIV infection in India	*JIAS*	No distinct HEU group; Age includes >5 years (4-15 years); Conference abstract;
**185**	2014	Sibiude, J. *et al*	Association between prenatal exposure to antiretroviral therapy and birth defects: an analysis of the French perinatal cohort study (ANRS CO1/CO11)	*PLoS Medicine*	No HEU group analysis;<br>Cohort spanned years <2000 (1994-2010).
**186**	2018	Siegle, C.B.H. & dos Santos Cardoso de Sa, C.	Concurrent validity between instruments of assessment of motor development in infants exposed to HIV	*Infant Behavior and Development*	No distinct HEU group – infants exposed to HIV which may include HIV-infected children
**187**	2014	Skeen, S. *et al*	Child development in HIV-positive and HIV-affected children in South Africa and Malawi-What role for community organisations?	*Children and Youth Services Review*	No specific HEU group analysis;<br>Age > 5years (4-13 years)
**188**	2010	Smith, L. *et al*	Neurological and neurocognitive function of HIV-infected children commenced on antiretroviral therapy	*South African Journal of Child Health*	No HEU group
**189**	2014	Smith, M.L. *et al*	Neurocognitive development in young HIV-Exposed uninfected children exposed pre-or perinatally to antiretroviral medications	*Canadian Journal of Infectious Diseases and Medical Microbiology*	Conference abstract; appears to be overlap with 2017 paper included in full text reviews
**190**	2015	Smith, M.L. *et al*	Neurocognitive outcomes in pre-school and early school-age HIV-exposed uninfected children exposed pre-or perinatally to antiretroviral medications	*Canadian Journal of Infectious Diseases and Medical Microbiology*	Conference abstract; appears to be overlap with 2017 paper included in full text reviews
**191**	2005	Smith, R.	Mental functioning of children with HIV infection: The preschool and early school-age years	*Dissertation Abstracts International*	Age >5 years included (3-7 years); Dissertation
**192**	2006	Smith, R. *et al*	Effects of perinatal HIV infection and associated risk factors on cognitive development among young children	*Pediatrics*	Cohort spanned years <2000 (1990-2000 births); Age >5 years (3-7 years)
**193**	2014	Stein, A. *et al*	Predicting long-term outcomes for children affected by HIV and AIDS: Perspectives from the scientific study of children's development	*AIDS*	Review
**194**	2016	Strehlau, R. *et al*	HIV-associated neurodevelopmental delay: prevalence, predictors and persistence in relation to antiretroviral therapy initiation and viral suppression	Child: care, health and development	No HEU group
**195**	2018	Strehlau, R. *et al*	Neurodevelopmental assessment of HIV-exposed uninfected and early-treated HIV-infected children: study protocol	*BMC research notes*	Protocol; no results
**196**	2019	Strehlau, R. *et al*	Interventions addressing neurodevelopmental delay in young children infected with or exposed to HIV: A scoping review	*Rehabilitation Oncology*	Review
**197**	2020	Strehlau, R. *et al*	A description of early neurodevelopment in a cohort of HIV-exposed uninfected children	*AIDS Care*	No HU control group or statistical comparison with normative data.
**198**	2019	Sudfeld, C.R. *et al*	Third trimester vitamin D status is associated with birth outcomes and linear growth of HIV-exposed uninfected infants in the United Statues	*JAIDS*	Focus on vitamin D exposure
**199**	2006	Tahan, T.T. *et al*	Neurological profile and neurodevelopment of 88 children infected with HIV and 84 seroreverter children followed from 1995 to 2002	*Brazilian Journal of Infectious Diseases*	Cohort spanned years <2000 (1995-2002) with no stratification
**200**	2005	Tardieu, M. *et al*	Cerebral MR imaging in uninfected children born to HIV-seropositive mothers and perinatally exposed to zidovudine	*American Journal of Neuroradiology*	Cohort spanned years <2000
**201**	2017	Tassiopouolos, K. *et al*	Blood lead levels and neurodevelopmental function in perinatally HIV-exposed, uninfected children in a U.S.-based longitudinal cohort study	*AIDS Research and Human Retroviruses*	Focus on lead exposure

**202**	2010	Thomaidis, L. *et al*	Cognitive and psychosocial development of HIV pediatric patients receiving highly active anti-retroviral therapy: A case-control study	*BMC Pediatrics*	No HEU group; Age >5years (range 3-18 years)
**203**	2001	Thompson, W.S. *et al*	Language, memory, and cognitive performance in minority children infected with HIV (immune deficiency)	*Dissertation Abstracts International*	Dissertation; Age >5 years (school age); No distinct HEU group (uninfected siblings and peers)
**204**	2018	Tomlinson, M. *et al*	Antenatal depressed mood and child cognitive and physical growth at 18-months in South Africa: a cluster randomized controlled trial of home visiting by community health workers	*Epidemiology and psychiatric sciences*	No distinct HEU group
**205**	2015	Torre, P. III *et al*	Hearing assessment data in HIV-infected and uninfected children of Cape Town, South Africa	*AIDS Care*	Age includes >5 years (4-14 years) and no sub-analysis
**206**	2015	Torre, P. III *et al*	Distortion product otoacoustic emission data in perinatally HIV-infected and HIV-exposed but uninfected children and adolescents in the Pediatric HIV/AIDS Cohort Study	*Pediatr Infect Dis J*	Age > 5 years (7-16 years)
**207**	2012	Torre, P. III *et al*	Hearing loss in perinatally HIV-infected and HIV-exposed but uninfected children and adolescents	*Pediatr Infect Dis J*	Age >5 years (7-16yrs)
**208**	2008	Urban, M.F. *et al*	Growth of infants born to HIV-infected women when fed a biologically acidified starter formula with and without probiotics	*S Afr J Clin Nutr*	Trial confounded outcome
**209**	2016	Van Dalen, Y.W. *et al*	Neurometabolite Alterations Associated With Cognitive Performance in Perinatally HIV-Infected Children	*Medicine*	No specified HEU group; Age >5 years (8-18 years)
**210**	2019	Van den Hof, M. *et al*	Lower IQ and poorer cognitive profiles in treated perinatally HIV-infected children is irrespective of having a background of international adoption	*PLOS ONE*	No specified HEU group; Age >5 years (mean 10.45 years)
**211**	2020	Van den Hof, M. *et al*	Neurocognitive development in perinatally human immunodeficiency virus-infected adolescents on long-term treatment, compared to healthy matched controls: a longitudinal study	*CID*	No specified HEU group; Age >5 years (8-18 years)
**212**	2016	Van Dyke, R.B. *et al*	The PHACS SMARTT study: Assessment of the safety of in utero exposure to antiretroviral drugs	*Frontiers in Immunology*	Review of SMARTT outcomes
**213**	2019	Vannappagari, V. *et al*	Pregnancy and neonatal outcomes following prenatal exposure to dolutegravir	*JAIDS*	No neurodevelopmental outcomes, reports birth defects
**214**	2017	Van Wyhe, K.S. *et al*	Cross-cultural assessment of HIV-associated cognitive impairment using the Kaufman assessment battery for children: a systematic review	*JIAS*	Review
**215**	2018	Visser, M.J. *et al*	A comparative study of the psychological problems of HIV-infected and HIV-uninfected children in a South African sample	*AIDS Care*	No HEU group; age >5 years (6-12 years)
**216**	2003	Von Giesen, H.J. *et al*	Delayed motor learning and psychomotor slowing in HIV-infected children	*Neuropediatrics*	No specified HEU group; Age >5 years (8-16 years)
**217**	2018	Wesevich, A. *et al*	PMTCT option b+ efavirenz and tenofovir exposure through breastfeeding and bayleys neurodevelopmental scores in Malawian infants	*Pediatrics*	No specified HEU group - infants born to HIV-positive mothers which may include HIV-infected children; Conference presentation
**218**	2019	White, M. & Connor, K.L.	Determining how in utero HIV exposure, with or without infection, influences neurodevelopment in infants before age three: Findings from an evidenced-based review of observational and experimental studies	*Reproductive Sciences Supplement*	Review; Conference presentation
**219**	2019	White, M. *et al*	Does the early nutritional environment and in utero HIV exposure, without infection, impact infant development?	*Reproductive Sciences Supplement*	Conference presentation; no HEU and neurodevelopment analysis results
**220**	2012	Whitehead, N.	The neurodevelopment of HIV positive infants on HAART compared to HIV exposed but uninfected infants	*NA*	Thesis.
**221**	2014	Whitehead, N *et al*	The neurodevelopment of HIV-infected infants on HAART compared to HIV-exposed but uninfected infants	*AIDS Care*	No HU control group or statistical comparison with normative data.
**222**	2010	Williams, P.L. *et al*	Neurodevelopment and in utero antiretroviral exposure of HIV-exposed uninfected infants	*Pediatrics*	Cohort spanned years <2000 (1993-2006)
**223**	2012	Williams, P.L. *et al*	A trigger-based design for evaluating the safety of in utero antiretroviral exposure in uninfected children of human immunodeficiency virus-infected mothers	*American Journal of Epidemiology*	Methods paper, no neurodevelopmental results
**224**	2016	Williams, P.L. *et al*	Antiretroviral exposure during pregnancy and adverse outcomes in HIV-exposed uninfected infants and children using a trigger-based design	*AIDS*	Age >5 years included.
**225**	2019	Williams, P.L. *et al*	Associations of maternal ARV use with microcephaly in HIV-exposed uninfected children	*Open Forum Infectious Diseases*	Conference presentation, paper included in full text
**226**	2017	Yadav, S.K. *et al*	Altered structural brain changes and neurocognitive performance in pediatric HIV	*NeuroImage*	No HEU group; Age >5 years (controls mean 11.2 years)
**227**	2019	Yang, L. *et al*	Child development in HIV exposed, uninfected children: Challenges with accessing services	*Paediatrics and Child Health*	Conference abstract; insufficient information on developmental outcomes

S6 Appendix: List of included studies and relevant articles

	Study name *(where available)*	Country	HEU v. HU articles	ART analysis articles	Secondary outcomes articles
1.	-	Canada	Alimenti *et al*., 2006		
2.	***Malaria RCT***	Uganda	Boivin *et al*., 2016		
3.	**PROMISE**	Malawi & Uganda	Boivin *et al*., 2019	Boivin *et al*., 2019	Aizire *et al*., 2020
4.	**Rakai Community Cohort Study** (RCCS); **Nevirapine (NVP) Prevention of Mother to Child HIV Transmission (PMTCT) study**	Uganda	Brahmbhatt *et al*., 2014		
5	**Tshipidi**	Botswana	Chaudhury *et al*., 2017	Chaudhury *et al*., 2018 (also Mma Bana)	
6	-	Brazil	Da Silva *et al*., 2017		
7	**PreNAPS** **PostNAPS**	Uganda	Familiar *et al*., 2018	Familiar *et al*., 2018	
8	-	Colombia	Gomez *et al*., 2009		Gomez *et al*., 2009
9	**HIVNET 012 clinical trial**	Zimbabwe	Kandawasvika *et al*., 2011		
10	-	Malawi	Landes *et al*., 2012		
11	**CHER**	South Africa	Laughton *et al*., 2012 Laughton *et al*., 2018		Laughton *et al*., 2012
12	**MCH-ART**	South Africa	Le Roux *et al*., 2018		Le Roux *et al*., 2019 (also HU2)
13	**Aluvia**	Zambia	Ngoma *et al*., 2014		
14	**SHINE**	Zimbabwe	Ntozini *et al*., 2020		
15	**SMARTT**	USA & Puerto Rico	Sirois *et al*., 2013	Caniglia *et al*., 2016 Rice *et al*., 2013 Rice *et al*., 2018 Sirois *et al*., 2013	Caniglia *et al*., 2016 Siberry *et al*., 2012 Jacobson *et al*., 2017 Williams *et al*., 2019
16	-	South Africa	Springer *et al*., 2012		Springer *et al*., 2012
17	**MIHS**	South Africa	Springer *et al*., 2018 Springer *et al*., 2020	Springer *et al*., 2018	Springer *et al*., 2018 Springer *et al*., 2020
18	-	Malawi	Struyf *et al*., 2019		
19	**-**	DRC	Van Rie *et al*, 2008 Van Rie *et al*, 2009		
20	**DCHS**	South Africa	Wedderburn *et al*., 2019		Donald *et al*., 2017 Tran *et al*., 2016
21	-	China	Wu *et al*., 2019		
22	**PMTCT RCT**	South Africa		Alcaide *et al*., 2019	
23	**Mpepu**	Botswana		Cassidy *et al*., 2019 (also Tshipidi and Mma Bana)	
24	**Mma Bana**	Botswana		Kacanek *et al*., 2018 (also Cassidy *et al*., 2019	
25	-	India		Rajan *et al*., 2017	
26	-	Canada		Smith *et al*., 2017	
27	-	Nigeria			Jumare *et al*., 2019
28	**CIGNIS**	Zambia			Filteau *et al*., 2011
29	-	USA			Neri *et al*., 2013
30	**Eunice Kennedy Shriver National Institute of Child Health and Human Development (NICHD) International Site Development Initiative (NISDI) study**	Latin America and Caribbean			Spaulding *et al*., 2016
31	-	Kenya			Pintye *et al*., 2015

S7 Appendix: Characteristics of studies examining neurodevelopment of HEU children compared to HU children

Author, year of publication, study	Country (time period)	Study design	Child age	N participants HEU	HU	HIV test in HEU children (age)	Maternal ART (%)	ART regimen	Infant PNP (duration)	Breastfeeding	Tool	Confounders included in adjusted analysis
Alimenti *et al.*, 2006[3]	Canada (2003 – 2004)	CS	18 – 36m	39	24	PCR (twice <6m) & Ab confirming seroreversion (6m)	100%	Triple therapy (quadruple in 3) AZT at delivery	AZT (neonatal period)	ND	BSID-II<br>VABS	Maternal substance use.
Boivin *et al.*, 2016[4]	Uganda (2010 – 2013)	Cohort / RCT	2yr<br>3yr	143<br>122	325<br>331	PCR at enrolment and (6w after cessation BF)	100%	Triple therapy	NVP (6wks)	100%; Duration HEU<HU	MSEL	Malaria, anaemia, age, WAZ, breastfeeding days, trial arm, sex, SES, observation days prior to randomization. Note RCT malaria intervention trial.
Boivin *et al.*, 2019[5]	Malawi and Uganda (2013 – 2014)	Cohort/ RCT	12, 24, 48, and 60m	405	456	PCR (12m, 24m)	54%<br>46%	Triple therapy<br>AZT	NVP (50%) None (50%) but maternal triple ART	HEU 73-83%, HU 95% 12m; Duration HEU<HU)	MSEL<br>KABC-II	HEU and HU children matched for age, sex, SES. Data collection site.
Brahmbhatt *et al*, 2014[6]	Uganda (not reported)	Cohort	0 to 6yr (median 36.1m HEU 57.5m HU)	105	108	PCR (<18m), EIA (>18 m)	ND	NVP	NVP (% ND)	ND	MSEL	Child age, weight and height.
Chaudhury *et al.*, 2017[7]	Botswana (2010 – 2012)	Cohort	2yr (22-29 m)	313 (BSID)<br>337 (DMC)	357 (BSID)<br>386 (DMC)	PCR (<18m) ELISA (18m)	36%<br>64%	Triple therapy<br>AZT	NVP (single dose) and AZT (1m)	HEU 9% HU 99.5% HEU<HU	BSID-III - adapted<br>DMC	Maternal age, income, education, and depression, household access to water, sanitation and electricity, food insecurity, housing type, cooking method. Prematurity & birthweight examined in sensitivity analyses.
Da Silva *et al.*, 2017[8]	Brazil (timeframe ND)	CS	4, 8, 12 & 18m	40 (10/age)	40 (10/age)	PCR (16m)	100%	Not specified	Not specified (6wks)	Interrupted	BSID-III	–
Familiar *et al.*, 2018[9]	Uganda (2012 – 2015)	LG	6 & 12m	75 complete data (79 total)	140 complete data (149 total)	DBS PCR	76%<br>24%	Triple therapy<br>None	ND	100%	MSEL	Child sex, maternal age, education level, marital status, asset index, employment status, social support and depression, HOME score. HAZ mediator.
Gomez *et al.*, 2009[10]	Colombia (not reported)	LG	0 – 24 m (3, 6, 9, 12 , 18 and 24m)	23	20	ELISA (18m)	100%	Triple therapy	ND	ND	BSID-II<br>DDST	–
Kandawasvika *et al.*, 2011[11]	Zimbabwe (2002 – 2004)	Cohort	3 to 12m (3m assessments)	188	287	PCR (<15m), Ab (>15mo)	In labour % ND	NVP	ND	99%	BINS	–
Landes *et al.*, 2012[12]	Malawi (2008)	Cohort	20m	128	200	Health passport, offered rapid testing	~10%<br>~70%	Triple therapy<br>NVP	NVP (66%)	HEU & HU>90% Duration HEU<HU	Milestones	–
Laughton *et al.*, 2012[13] **CHER**	South Africa (2005 – 2006)	Cohort	11m (10-16m) 11.5m HEU 11.5m HU)	28	34	HEU: PCR at baseline and 1 month after PCV 3rd dose (12-24 wks).	ND	ART/PMTCT prophylaxis - personal correspondence	NVP and AZT (7 days) (%ND)	ND	GMDS	–
Laughton *et al.*, 2018[14] **CHER**	South Africa (2005 – 2013)	Cohort	11 to 60m<br>(11, 20, 30, 42, 60 m)	34	39	HEU: PCR at baseline and 1 month after PCV 3rd dose (12-24 wks).	85%<br>15%	PMTCT (mainly NVP and AZT)<br>Unknown	NVP and AZT (85%)	ND	GMDS<br>VMI	–
Le Roux *et al.*, 2018 [15]	South Africa (2013 – 2016)	Cohort	13m (IQR 12-14)	215	306	PCR (12m)	100%	Triple therapy (TDF+FTC+ EFV)	NVP +/- AZT	100% (duration HEU<HU)	BSID-III (no receptive language)<br>OFC	Gestation, sex, SGA, Maternal education, intimate partner violence, Risky drinking, breastfeeding duration, housing, maternal age, employment; planned pregnancy; postpartum depression
Ngoma *et al.*, 2014[16]	Zambia (2011 – 2013)	Double cohort	15 to 36m Mean HEU 22.4m HU 24.1	97	103	RNA PCR on DBS; Controls had serology performed.	100%<br>In labour	Triple therapy (ZDV+3TC+ LPV/r)<br>NVP in labour	NVP (48h) and AZT (1wks)	100% (duration HEU<HU)	FSDQ (CAT/CLAMS)	Child age and birthweight, maternal education, monthly income.

Ntozini *et al*., 2020[17]	Zimbabwe (2016 – 2017)	RCT	24m	205	1175	PCR / Rapid (18m)	86%<br><br><br>24%	ART (majority TDF-based triple therapy)<br>Unknown	ND	100%	MDAT<br>CDI<br>A-not-B	(1): Child age, sex and month of birth, trial arm, study nurse.<br>(2): maternal education, household wealth, maternal age, parity.
Sirois *et al*., 2013[18]	USA & Puerto Rico (2007 – 2011)	Cohort	12.7m (9 to 15m)	374	49	ND in paper; study design on clinicaltrials.gov	97%<br>2%<br>1%	Triple therapy<br>Other<br>None	AZT (8w) 97%	ND	BSID-III	–
Springer *et al*., 2012[19]	South Africa (2009) (Pilot study)	CS	17 to 19m	17	20	PCR (2, 6 & 12wks)	94%	ART/ PMTCT prophylaxis	ND	HEU 6%, HU 100%	GMDS<br>OFC	–
Springer *et al*., 2018[20] **MIHS**	South Africa (2012 – 2013)	Nested cohort	12m (11-14)	58	38	ELISA with PCR if positive (12m)	50%<br>50%	cART<br>AZT monotherapy	NVP and AZT (1wks)	HEU 12%, HU 66% at 6m	BSID-III<br>ADBB<br>OFC	–
Springer *et al*., 2020[21] **MIHS**	South Africa (2012 – 2013)	Cohort	3yrs (30-42 m)	32	27	ELISA with PCR if positive (12m)	41%<br>59%	cART<br>AZT	NVP and AZT (1wks)	HEU 9%, HU 67% at 6m	BSID-III<br>SDQ<br>OFC	Stunting, maternal education.
Struyf *et al*., 2019[22]	Malawi (2008 – 2011)	Cohort	15wks to 24m (BSID at 15w, 6, 9, 12, 15, 18 and 24m)	289	170	PCR (6w and follow up visits), Ab (>18mo) also for HU. Note 21/289 seroconverted and were censored from analysis	29.5%	NVP only	NVP (90%)	ND	BSID-III<br><br>Cognitive only	–
Van Rie et al, 2008[23] **(DRC cohort)**	Democratic Republic of Congo (DRC) (2004-2005)	CS	18m to 72m (HEU median 33.4 m; HU median 45.6m)	35	90	HIV-uninfected children who were orphans of maternal AIDS / whose mothers had symptomatic AIDS	ND	Unclear but ART was available at the time	ND	ND	BSID (18-29m)<br>PDMS<br>SONR (30-72m)<br>RITLS (18-36m)	–
Van Rie *et al*., 2009[24] **(DRC cohort)**	DRC (2004 – 2005)	Cohort	18m to 71m	35	90	*As above.* ELISA available.	ND	Unclear but ART was available at the time	ND	ND	BSID-II<br>PDMS<br>SONR	–
Wedderburn *et al*., 2019[25]	South Africa (2012 – 2015)	Cohort	6m	61	199	PCR at 6 weeks, PCR/Ab (9m/18m)	88%	Triple therapy	NVP 87%; NVP+AZT 13%	HEU 14%, HU 18% at 6m. Duration HEU<HU	BSID-III	Child age and sex, maternal age and education, household income.<br>Sensitivity analysis prematurity, depression, breastfeeding.
			2yr	168	564		12%	AZT				
Wu *et al*., 2019[26]	China (2010 – 2013)	CS	6m to 3yr in five age bands	250 (50/age)	250 (50/age)	PCR (6wks)	100%	Triple therapy	AZT or NVP (4 to 6w) (100%)	ND but guidelines HEU FF (~97.9%)	BSID-III	HEU and HU children matched for child gender, age, maternal age and residency. Neonatal jaundice, child anaemia, low birthweight, prematurity and malnutrition, maternal education and smoking.

Abbreviations | ND: not documented; HEU: children who are HIV-exposed and uninfected; HU: children who are HIV-unexposed; CS: cross-sectional study; LG: longitudinal study; RCT: randomized controlled trial; DRC: Democratic Republic of Congo; yr: year; m: months; wks: weeks; ART: antiretroviral treatment; PNP: postnatal prophylaxis; AZT: zidovudine; 3TC: lamivudine; NVP: nevirapine; LPV/r: lopinavir/ritonavir; TDF: tenofovir: FTC: emtricitabine; EFV: efavirenz; cART: combination ART; PMTCT: prevention of mother-to-child transmission; Ab: HIV antibody test; PCR: HIV polymerase chain reaction test; DBS: Dried blood spot; FF: formula feeding; OFC: occipitofrontal circumference; HCAZ: head circumference-for-age; NP: neonatal period; WAZ: weight for age; SES: socioeconomic status; SGA: small for gestational age; STI: sexually transmitted infection; All assessment tool abbreviations are listed in S8 Appendix.

S8 Appendix: Child development tools used across included studies highlighting those in the meta-analysis

Tool	Abbreviation	Scales	Domains included in meta-analysis
A-not-B task	A-not-B	Object permanence; early executive	
Alarm Distress Baby Scale	ADBB	Socioemotional state	
Ages & Stages Questionnaire	ASQ	Cognitive, language, motor,	
Bayley Infant Neurodevelopmental Screener	BINS	Risk for developmental delay	
Bayley Scales of Infant & Toddler Development, 2nd edition	BSID-II	Mental & psychomotor development	
Bayley Scales of Infant & Toddler Development, 3rd edition	BSID-III	Cognitive, receptive language, expressive language, fine motor, gross motor, social-emotional,	Cognitive, receptive language, expressive language, fine motor, gross motor
Beery-Buktenica developmental tests of Visual Motor Integration	VMI	Visual perception, motor, and hand-eye coordination	
Capute Scales Clinical Adaptive Test: Cognitive Adaptive Test	CAT	Cognition, visual-motor	
Capute Clinical Linguistic and Auditory Milestone Scales	CLAMS	Language and nonverbal problem-solving skills	
Denver Developmental Screening Test	DDST	Gross motor, language, fine motor, adaptive, and personal-social,	
Development Assessment Scale for Indian Infants	DASII	Motor and mental scales	
Developmental Milestones Checklist	DMC	Locomotor, fine motor, language, personal-social	
Dubowitz Neonatal Neurobehavioral Tool	DNNT	Neurobehaviour and neurological	
Full-Scale Developmental Quotient	FSDQ	Cognition, Language, Social	
Goldman-Fristoe Test of Articulation	GFTA	Language	
Griffiths Mental Development Scales	GMDS	Locomotor, personal-social, hearing & language, eye & hand coordination, performance	
Head circumference-for-age z score or Occipital frontal circumference	HCAZ or OFC		
Kaufman Assessment Battery for Children, 2nd edition	KABC-II	Simultaneous, sequential, learning, planning, knowledge	
MacArthur-Bates Communicative Developmental Inventories	CDI	Language by parent report	CDI vocabulary used for expressive language per correspondence with expert
Malawi Developmental Assessment Tool	MDAT	Total score, gross motor, fine motor, language, social	Total score, gross motor, fine motor, language<br>- MDAT language used for receptive language per correspondence with expert; total score used to represent cognitive development
Mullen Scales of Early Learning	MSEL	Visual reception, fine motor, gross motor, receptive and expressive language, composite	Fine motor, gross motor, receptive and expressive language, composite<br>- Early learning composite used to represent cognitive development
Peabody Developmental Motor Scales	PDMS	Motor	
Peabody Picture Vocabulary Test	PPVT	Language	
Personal, social and emotional development	PSED	Social-emotional development	
Rossetti Infant-Toddler Language Scale	RITLS	Language	
Strengths and Difficulties Questionnaire	SDQ	Socio-emotional	
Snijders-Oomen Nonverbal Intelligence Test	SONR	Intelligence	
Test of early language development	TELD	Receptive and expressive language	
Vineland Adaptive Behaviour Scales	VABS	Daily living, socialization, communication, motor	
WHO Milestones Chart	Milestones	Motor, language	
Wechsler Preschool and Primary Scales of Intelligence	WPPSI	Verbal IQ, Performance IQ, Full Scale IQ and General Language	

S9 Appendix: Quality assessment and risk of bias of studies comparing HEU and HU children

Study authors	Year	1. Objective stated	2. Study population defined and representative*	3. Participation rate is >50%*	4. Control subjects are from the same community*	5a. Sample size calculation	5b. Sample is >50 subjects per group	6. Exposure is measured before outcome#	7. Timeframe is sufficient to see the exposure effects#	8. Additional analyses performed (ART/VL/CD4)	9. A valid HIV test is used in mothers	10. A valid HIV test is used in children	11. Valid neurodevelopment tests are used in children**	12. Assessors are blinded to child HIV exposure status**	13. Lost to follow up is <20%^	14. Potential confounders adjusted for (or matched)#	Quality assessment
Alimenti *et al.*	2006	✓	✓	?	✗	✓	✗	✗	✗	✓	✓	✓	✓	✓	NA	✓	**FAIR**
Boivin *et al.*	2016	✓	?	✓	✓	✗	✓	✓	✓	✗	✓	✓	✓	?	✗	✓	**FAIR**
Boivin *et al.*	2019	✓	✓	✓	✓	✗	✓	✓	✓	✓	✓	✓	✓	?	✓	✓	**GOOD**
Brahmbhatt *et al.*	2014	✓	✗	?	?	✗	✓	✓	✓	✗	✓	✓	✓	?	NA	✓	**FAIR**
Chaudhury *et al.*	2017	✓	✓	✓	✓	✓	✓	✓	✓	✓	✓	✓	✓	?	✓	✓	**GOOD**
da Silva *et al.*	2017	✓	✗	?	✓	✓	✗	✗	✗	✗	?	✓	✓	?	NA	✗	**POOR**
Familiar *et al.*	2018	✓	✓	✓	✓	✓	✓	✓	✓	✓	✓	✓	✓	?	?	✓	**GOOD**
Gomez *et al.*	2009	✓	✗	✗	?	✗	✗	✓	✓	✗	✓	✓	✓	?	✗	✗	**POOR**
Kandawasvika *et al.*	2011	✓	?	✓	?	✓	✓	✓	✓	✗	✓	✓	?	✓	?	✗	**FAIR**
Landes *et al.*	2012	✓	✓	✗	✓	✗	✓	✓	✓	✗	?	✓	?	?	✓	✗	**POOR**
Laughton *et al.*	2012	✓	✗	✗	✓	✗	✗	✓	✓	✗	✓	✓	✓	✓	NA	✗	**FAIR**
Laughton *et al.*	2018	✓	✗	✓	✓	✓	✗	✓	✓	✗	✓	✓	✓	✓	✗	✗	**FAIR**
le Roux *et al.*	2018	✓	✓	✓	✓	✓	✓	✓	✓	✗	✓	✓	✓	?	✗	✓	**GOOD**
Ngoma *et al.*	2014	✓	?	✓	✗	✗	✓	✗	✗	✗	✓	✓	✓	?	NA	✓	**FAIR**
Ntozini *et al.*	2020	✓	✓	✗	✓	✗	✓	✓	✓	✗	✓	✓	✓	?	✓	✓	**GOOD**
Sirois *et al.*	2013	✓	✓	✓	✓	✗	✗	✓	✓	✓	✓	✓	✓	?	✗	✗	**GOOD**
Springer *et al.*	2012	✓	✓	?	✓	✗	✗	✗	✗	✗	✓	✓	✓	✓	✗	✗	**FAIR**
Springer *et al.*	2018	✓	✓	✓	✓	✓	✗	✓	✓	✓	✓	✓	✓	✓	✗	✗	**GOOD**
Springer *et al.*	2020	✓	✓	✗	✓	✗	✗	✓	✓	?	✓	✓	✓	✓	✗	?	**FAIR**
Struyf *et al.*	2019	✓	✓	?	✓	✓	✓	✓	✓	✓	✓	?	✓	?	✗	✗	**FAIR**
van Rie *et al.*	2008	✓	✗	?	✗	✗	✗	✗	✗	✗	✗	?	✓	?	NA	✗	**POOR**
van Rie *et al.*	2009	✓	✗	?	✗	✗	✗	✓	✓	✗	✗	?	✓	?	?	✗	**POOR**
Wedderburn *et al.*	2019	✓	✓	?	✓	✗	✓	✓	✓	✓	✓	✓	✓	✓	?	✓	**GOOD**
Wu *et al.*	2019	✓	✓	✗	?	✗	✓	✗	✗	✗	✓	✓	✓	?	NA	✓	**FAIR**

✓ Yes; ✗ No; ? Not reported / Not documented / Cannot determine; NA: not applicable; ART: antiretroviral therapy; VL: viral load; CD4: CD4 cell count.
Types of bias: *Selection bias; **Information bias; ^Attrition bias; #Confounding

S10 Appendix: Characteristics of studies of HEU children comparing different maternal ART regimens

Author, year of publication	Country (time period)	Study design	Child age	Tool	Last HIV test (age)	Maternal ART regimen (Group A; *N*)	Maternal ART regimen (Group B; *N*)	Maternal ART regimen/control (Group C; *N*)	Infant PNP (duration)	Breast-feeding	Confounders included in adjusted analysis
Alcaide *et al*., 2019[27]	South Africa (2015 – 2018)	CS	9 to 20 m<br>13.4±1.9 HEU	BSID-III	PCR from DBS (12m)	Triple therapy, EFV/TDF/FTC detectable at 32 weeks' gestation<br>(66)	Triple therapy, EFV/TDF/FTC undetectable<br>(14)	-	ND	ND	Intimate partner violence, maternal depression.
Boivin *et al*., 2019*[5]	Malawi and Uganda (2013 – 2014)	Cohort / RCT	12m,<br>2, 4 & 5yr	MSEL<br>KABC-II	PCR (12, 24 m)	Group 1 & 2<br>Antenatal triple ART (PI-based)<br>+ postnatal triple ART or infant NVP<br>(93 / 103)	Groups 3 & 4<br>Antenatal AZT monotherapy<br>+ postnatal triple ART or infant NVP<br>(88 / 80)	HU<br>(374)	*See groups*	Yes	Data collection site.<br>*Note:* PI-based regimen: LPV/r + 3TC + AZT or LPV/r + FTC + TDF
Caniglia *et al*., 2016[28] **SMARTT**	USA & Puerto Rico (2006 – 2013)	Cohort	9 to 15m	BSID-III<br>OFC	ND	ART: ATV-based<br>1st trimester<br>2nd/3rd trimester<br>(Total N= 575)	ART: no ATV<br>1st trimester<br>2nd/3rd trimester	-	ND	ND	Maternal education, CD4, HIV RNA, year, illicit substances, alcohol, tobacco, race, ethnicity, primary language, household income, age, Full Scale IQ, STI, LBW, GA.
Cassidy *et al*., 2019[29] **Tshipidi plus (Mpepu; Mma Bana; Tshipidi)**	Botswana (2016 – 2017)	Cohort	2yr	BSID-III<br>DMC<br>PSED	Per individual cohort	Triple therapy, EFV/TDF/FTC<br>(126)	Triple therapy, non-EFV-multiple<br>(367)	-	NVP (single dose) and AZT (1m) or NVP(1m)	EFV 29%; Not EFV 73%	Child age, sex, feeding method, *in utero* ART initial exposure timing, maternal age, employment, income, marital status, indoor faucet, electricity, indoor toilet.<br>Sensitivity analyses: Preterm birth, LBW.
Chaudhury *et al*., 2018[30]<br>**Mma Bana**<br>**Tshipidi**	Botswana<br>(2006 – 2008)<br>(2010 – 2012)	Cohort & RCT	2yr	BSID-III<br>DMC	PCR at birth, 1, 6, 12 months. ELISA (>18mo)	ART: multiple regiments<br>(382)	AZT monotherapy<br>(210)	-	NVP (single dose) and AZT (1m)	ART 71.5%<br>AZT 8.1%	Maternal age, education, income, CD4 cell count, year of neurodevelopmental testing. Sensitivity analysis restricted to formula fed infants from one cohort. Prematurity & birthweight examined in sensitivity analyses.
Familiar *et al*., 2018*[9]	Uganda (2012 – 2015)	Cohort	6 & 12m	MSEL	DBS PCR	ART: multiple regimens<br>(57)	No therapy<br>(18)	HU<br>(140)	Not specified	Yes	Child age and sex, maternal age, education level, marital status, HOME, SES, social support and depression.
Kacanek *et al*., 2018[31] **Mma Bana**	Botswana (2006 – 2008)	CS/RCT	2yr	BSID-III<br>DMC<br>PSED	Per study: birth, 1m, 6m PCR	Triple NRTI<br>ABC/AZT/3TC<br>(101)	Dual NRTI+PI<br>LVP/r / AZT/3TC<br>(96)	-	NVP (single dose) and AZT (1m)	Yes	Low maternal body mass index at follow-up, child age, access to electricity in the home, GA at enrolment.<br>Randomised from 26-34w gestation.
Rajan et al, 2017[32]	India<br>(2013 – 2015)	Cohort	6-18 m<br>(assessed at enrolment, 3m, 6m)	DASII	PCR (6 wks, 6 m, and 12 wks after breast feeding); Serology at 18 m	ART<br>(31)	No ART<br>(10)	-	NVP (single dose n=80), NVP (6 wks, n=30), none (n=3)	ND	-

Rice<br>*et al.*, 2013[33]<br>**SMARTT**	USA & Puerto Rico<br>(2007 – 2011)	Cohort	1 yr<br><br>2 yr	CDI<br><br>ASQ	ND	cART<br>PI regimen<br>NNRTI<br>(Total N=535 at 12m)<br>(Total N=503 at 24m)<br>Individual drugs<br>(464 at 12m)<br>(431 at 24m)	Non-cART<br>No PI<br>No NNRTI	-	AZT-alone; combination	ND	Child sex, age and language exposure, neonatal prophylaxis, maternal IQ, CD4 cell count, viral load during pregnancy, caregiver health problems.
Rice<br>*et al.*, 2018[34]<br>**SMARTT**	USA & Puerto Rico<br>(2007 – 2011)	Cohort	3 & 5yr	Speech: GFTA<br>Language: TELD<br>Language: PPVT	ND	Individual drugs<br>(208 at 3y)<br>(429 at 5y)<br><br>Combination ART	 <br><br>Triple NRTI	-	ND	ND	Child age, sex, race and ethnicity, maternal education, caregiver health problems and alcohol use during pregnancy.
Sirois<br>*et al.*, 2013*[18]<br>**SMARTT**	USA & Puerto Rico<br>(2007 – 2011)	Cohort	12m<br>(9 to 15m)	BSID-III	ND	Combination ART (>=3 drugs >= 2 classes)<br>PI-containing (with or without NNRTI)<br>NNRTI-containing (without PI)<br>Individual drugs<br>N=374	Non-combination ART<br><br>NRTI only<br>NRTI only		AZT (8wks) 97%	ND	Child age, year of delivery, sex, last viral load prior to delivery, STI during pregnancy, maternal full scale IQ score, substance use, maternal age at delivery.<br>Sensitivity analyses: prematurity and SGA
Smith<br>*et al.*, 2017[35]	Canada<br>ND	LG	3.5yr (5.5 yr not included in age range)	WPPSI<br>VABS<br>VMI	2+ DNA PCR assays or after 1 month negative HIV serology at any age	Triple therapy, PI-based<br>(43)	Triple therapy, NNRTI-based<br>(16)	-	AZT<br>+/- NVP, 3TC	ND	-
Springer<br>*et al.*, 2018*[20]	South Africa<br>(2012 – 2013)	Cohort	12m<br>(11 to 14m)	BSID-III<br>ADBB<br>OFC	ELISA with PCR if positive (12m)	ART: multiple regimens<br>(29)	AZT monotherapy<br>(29)	HU<br>(38)	NVP and AZT (1wks)	HEU 12% 6m, ND by ART	–

Abbreviations | ND: not documented; HEU: children who are HIV-exposed and uninfected; HU: children who are HIV-unexposed; CS: cross-sectional study; LG: longitudinal study; RCT: randomized controlled trial; yr: year; m: months; wks: weeks; PCR: HIV polymerase chain reaction test; DBS: Dried blood spot; ART: antiretroviral treatment; PNP: postnatal prophylaxis; AZT: zidovudine; 3TC: lamivudine; NVP: nevirapine; LPV/r: lopinavir/ritonavir; TDF: tenofovir: FTC: emtricitabine; EFV: efavirenz; ATV: atazanavir; ABC: abacavir; NRTI: nucleoside reverse transcriptase inhibitor; NNRTI: non-nucleoside reverse transcriptase inhibitor; PI: protease inhibitor; cART: combination ART; PMTCT: prevention of mother-to-child transmission; FF: formula feeding; NP: neonatal period; WAZ: weight for age; LBW: low birth weight; SES: socioeconomic status; GA: Gestational age; STI: sexually transmitted infection; All assessment tool abbreviations are listed in S8 Appendix.

*Also in HEU v. HU analysis

S11 Appendix: Characteristics of studies examining head circumference and neuroimaging

Author, year of publication	Country (time period)	Study design	Child age	N HEU	N HU	HIV test in HEU children (age)	Maternal ART (%)	Maternal ART regimen	Infant PNP (duration)	Breast-feeding	Confounders included in adjusted analysis
**Aim 1: HEU children versus HU children - Head circumference**											
Donald *et al.*, 2017[36] **DCHS**	South Africa (2012 – 2015)	Cohort	Neonatal period	131	536	PCR at 6 weeks, PCR/Ab (9m/18m)	100%	Triple ART or AZT monotherapy	NPV or NVP+AZT	ND	Socioeconomic status, depression, smoking, alcohol use in interaction variables (HIV by alcohol use and HIV by smoking status).
Le Roux *et al.*, 2019[37]	South Africa (2013 – 2016)	Cohort	Birth to 1yr (0, 3m, 6m, 9m, 12m)	461	411	PCR at 6 weeks and 48 weeks.	100%	Triple therapy	ND	Yes	Age-adjusted, sex-adjusted and gestation-adjusted z-scores. Socioeconomic factors, risky drinking, intimate partner violence, recent childhood illness, and infant feeding) with and without adjustment for birth outcomes.
Jumare *et al.*, 2019[38]	Nigeria (2013 – 2017)	Cohort	Birth to 18m	297	103	PCR at birth, 3-4wks, 6wks, 1yr or 6wks postbreastfeeding	100%	Triple therapy	NVP (6w)	Yes	WHO standard z-scores. Maternal education, marital status, breastfeeding, prematurity, maternal weight and baseline Z-score.
Gomez *et al.*, 2009*[10]	Colombia (not reported)	LG	Birth to 2yrs (0, 3, 6m, 9m, 12m, 18m, 2y)	23	20	ELISA (18m)	100%	Triple therapy, multiple	ND	ND	-
Filteau *et al.*, 2011[39]	Zambia (2005 – 2009)	RCT	6m to 2 yrs	125	382	Ab testing at 18m	ND	NVP	NVP	HEU 42%, HU 97%	- (Only baseline characteristics included here)
Neri *et al.*, 2013[40]	USA (2006 – 2009)	LG	2w to 2yrs (mean age 10m)	111 (82 in matched group)	82	PCR first 6 m, Ab after 12m	96%	Triple therapy	AZT (6w)	ND	Age, sex, race.
Springer *et al.*, 2018*[20] **MIHS**	South Africa (2012 – 2013)	Cohort	12m (11-14)	58	38	ELISA with PCR if positive (12m)	50%<br>50%	cART<br>AZT	ND	HEU no (12%), HU yes (66% at 6 months)	-
Aizire *et al.*, 2020[41]	Malawi and Uganda (2013 – 2014)	Cohort	1 to 2yrs (1yr, 2yr)	471	462	Documentation of uninfected status	54%<br>45%<br>1%	Triple therapy<br>AZT<br>No ART	NVP in some	HEU Yes, HU Yes. HU>HEU	Breastfeeding status, maternal age, electricity/gas use and tap-water use.
Laughton *et al.*, 2012*[13]	South Africa (2005 – 2006)	Cohort	11m (10-16m) 11.5m HEU 11.5m HU)	28	34	HEU: PCR at baseline and 1 month after PCV 3rd dose (12-24wks).	ND	NVP and AZT	ND. Likely NVP and AZT	ND	-
Springer *et al.*, 2012*[19]	South Africa (2009)	CS	17 to 19m	17	20	PCR (2, 6 & 12wks)	94%	PMTCT prophylaxis or cARV	ND	HEU no, HU yes	-
Springer *et al.*, 2020*[21] **MIHS**	South Africa (2012 – 2013)	Cohort	2 to 3yr (30 to 42m)	32	27	ELISA with PCR if positive (12m)	40.6%<br>59.4%	cART<br>AZT	NVP and AZT (1w)	HEU low (9%), HU (67%) at 6 months	-

**Aim 1: HEU children versus HU children - Neuroimaging**											
Tran *et al.*, 2016[42] **DCHS**	South Africa (2012 – 2015)	Cohort	Neonatal period	15	22	PCR at 6 weeks, PCR/Ab (9m/18m)	100%	Triple ART	NVP / NVP & AZT	ND	Neonatal postnatal age and infant sex.
**Aim 2: ART analyses - Head circumference**				**Group A**	**Group B**						
Spaulding *et al.*, 2016[43]	Latin America and Caribbean (2002 – 2009)	Cohort	Birth to 6m (0-3m, 6m)	1400 Multiple ART	-	HIV virologic assay at 1m and 4m or older or HIV Ab after 6 m	67% 32% 2%	PI + 2 NRTIs<br>2 NRTIs + NNRTI<br>*Other/non-adherent*	AZT	ND; likely none	Maternal and infant demographics and delivery-, infant-, and maternal HIV-, and infant HIV treatment-related covariates including congenital and infant infections, obstetric complications, and maternal infections were considered.
Pintye *et al.*, 2015[44]	Kenya (2013)	CS	6w and 9m	TDF+ 51	TDF- 104	PCR testing	100%	Triple therapy, multiple	ND	Yes	Maternal age, education level, time since HIV diagnosis, infant breastfeeding, gestational age, maternal WHO clinical stage, timing of ART initiation, trimester of first use of 3-drug combination ART and PI-containing ART regimen.
Siberry *et al.*, 2012[45] **SMARTT**	USA (2005-2010)	Cohort	Birth and 1yr	TDF+ 274 209	TDF- 416 361	ND	100%	Triple therapy, multiple	ND	No	Infant sex, household income, maternal viral load, maternal tobacco use during pregnancy, birth cohort, race, gonorrhoea infection.<br>Sensitivity analysis including GA.
Caniglia *et al.*, 2016[28] ***SMARTT***	USA & Puerto Rico (2006 – 2013)	Cohort	1yr	ART: ATV+ N=127	ART: ATV- N=525	ND	100%	ART, multiple	ND	ND	Maternal education, CD4, HIV RNA, year, illicit substances, alcohol, tobacco, race, ethnicity, primary language, household income, age, Full Scale IQ, STI, LBW, GA, trimester of ART initiation.
Jacobson *et al.*, 2017[46] **SMARTT**	USA & Puerto Rico (2007 – 2011)	Cohort	2yr	509 Multiple ART	–	ND	100%	Triple therapy, multiple	ND	ND	Region, alcohol, tobacco, income, language at home.
Williams *et al.*, 2020[47] **SMARTT**	USA & Puerto Rico (2007 – 2017)	Cohort	Birth to 5yrs	3055 Multiple ART	-	ND	141 2842	EFV+<br>non-EFV+<br>Multiple combinations assessed	ND	ND	Education, household income, alcohol use during pregnancy, birth cohort.

Abbreviations | ART: antiretroviral treatment; AZT: zidovudine; EFV: efavirenz; TDF: tenofovir; NVP: nevirapine; 3TC: lamivudine; FTC: emtricitabine; cART: combination ART; PI: protease inhibitor; NRTI: nucleoside reverse transcriptase inhibitor; NNRTI: non-nucleoside reverse transcriptase inhibitor; BF: breastfeeding; NP: neonatal period; PNP: postnatal prophylaxis; HEU: Children who are HIV-exposed and uninfected; HU: Children who are HIV-unexposed; CS: cross-sectional study; LG: longitudinal study; RCT: randomized controlled trial; HCAZ: head-circumference-for-age z-score; ND: not documented

References:

1. WHO. New data on the prevention of mother-to-child transmission of HIV and their policy implications : conclusions and recommendations : WHO Technical consultation on behalf of the UNFPA/UNICEF/WHO/UNAIDS Inter-Agency Task Team on Mother-to-Child Transmission of HIV. Geneva, 2001.
2. Villagomez AN, Munoz FM, Peterson RL, et al. Neurodevelopmental delay: Case definition & guidelines for data collection, analysis, and presentation of immunization safety data. *Vaccine* 2019; **37**(52): 7623-41.
3. Alimenti A, Forbes JC, Oberlander TF, et al. A prospective controlled study of neurodevelopment in HIV-uninfected children exposed to combination antiretroviral drugs in pregnancy. *Pediatrics* 2006; **118**(4): e1139-45.
4. Boivin MJ, Sikorskii A, Familiar-Lopez I, et al. Malaria illness mediated by anaemia lessens cognitive development in younger Ugandan children. *Malar J* 2016; **15**: 210.
5. Boivin MJ, Maliwichi-Senganimalunje L, Ogwang LW, et al. Neurodevelopmental effects of ante-partum and post-partum antiretroviral exposure in HIV-exposed and uninfected children versus HIV-unexposed and uninfected children in Uganda and Malawi: a prospective cohort study. *The lancet HIV* 2019; **6**(8): e518-e30.
6. Brahmbhatt H, Boivin M, Ssempijja V, et al. Neurodevelopmental benefits of antiretroviral therapy in Ugandan children aged 0-6 years with HIV. *Journal of acquired immune deficiency syndromes* 2014; **67**(3): 316-22.
7. Chaudhury S, Williams PL, Mayondi GK, et al. Neurodevelopment of HIV-Exposed and HIV-Unexposed Uninfected Children at 24 Months. *Pediatrics* 2017; **140**(4).
8. da Silva KM, de Sa CD, Carvalho R. Evaluation of motor and cognitive development among infants exposed to HIV. *Early Hum Dev* 2017; **105**: 7-10.
9. Familiar I, Collins SM, Sikorskii A, et al. Quality of Caregiving is Positively Associated With Neurodevelopment During the First Year of Life Among HIV-Exposed Uninfected Children in Uganda. *Journal of acquired immune deficiency syndromes* 2018; **77**(3): 235-42.
10. Gomez C, Archila ME, Rugeles C, Carrizosa J, Rugeles MT, Cornejo JW. [A prospective study of neurodevelopment of uninfected children born to human immunodeficiency virus type 1 positive mothers]. *Revista de neurologia* 2009; **48**(6): 287-91.
11. Kandawasvika GQ, Ogundipe E, Gumbo FZ, Kurewa EN, Mapingure MP, Stray-Pedersen B. Neurodevelopmental impairment among infants born to mothers infected with human immunodeficiency virus and uninfected mothers from three peri-urban primary care clinics in Harare, Zimbabwe. *Developmental medicine and child neurology* 2011; **53**(11): 1046-52.
12. Landes M, van Lettow M, Chan AK, Mayuni I, Schouten EJ, Bedell RA. Mortality and health outcomes of HIV-exposed and unexposed children in a PMTCT cohort in Malawi. *PloS one* 2012; **7**(10): e47337.
13. Laughton B, Cornell M, Grove D, et al. Early antiretroviral therapy improves neurodevelopmental outcomes in infants. *Aids* 2012; **26**(13): 1685-90.
14. Laughton B, Cornell M, Kidd M, et al. Five year neurodevelopment outcomes of perinatally HIV-infected children on early limited or deferred continuous antiretroviral therapy. *Journal of the International AIDS Society* 2018; **21**(5): e25106.
15. le Roux SM, Donald KA, Brittain K, et al. Neurodevelopment of breastfed HIV-exposed uninfected and HIV-unexposed children in South Africa. *Aids* 2018; **32**(13): 1781-91.
16. Ngoma MS, Hunter JA, Harper JA, et al. Cognitive and language outcomes in HIV-uninfected infants exposed to combined antiretroviral therapy in utero and through extended breast-feeding. *Aids* 2014; **28 Suppl 3**: S323-30.
17. Ntozini R, Chandna J, Evans C, et al. Early child development in children who are HIV-exposed uninfected compared to children who are HIV-unexposed: observational sub-study of a cluster-randomized trial in rural Zimbabwe. *Journal of the International AIDS Society* 2020; **23**(5): e25456.
18. Sirois PA, Huo Y, Williams PL, et al. Safety of perinatal exposure to antiretroviral medications: developmental outcomes in infants. *The Pediatric infectious disease journal* 2013; **32**(6): 648-55.
19. Springer P, Laughton B, Tomlinson M, Harvey J, Esser M. Neurodevelopmental status of HIV-exposed but uninfected children: A pilot study. *SAJCH South African Journal of Child Health* 2012; **6**(2): 51-5.
20. Springer PE, Slogrove AL, Laughton B, et al. Neurodevelopmental outcome of HIV-exposed but uninfected infants in the Mother and Infants Health Study, Cape Town, South Africa. *Tropical medicine & international health* 2018; **23**(1): 69-78.
21. Springer PE, Slogrove AL, Kidd M, et al. Neurodevelopmental and behavioural outcomes of HIV-exposed uninfected and HIV-unexposed children at 2-3 years of age in Cape Town, South Africa. *AIDS care* 2020; **32**(4): 411-9.
22. Struyf T, Dube Q, Cromwell EA, Sheahan AD, Heyderman RS, Van Rie A. The effect of HIV infection and exposure on cognitive development in the first two years of life in Malawi. *European journal of paediatric neurology* 2020; **25**: 157-64.

23. Van Rie A, Mupuala A, Dow A. Impact of the HIV/AIDS epidemic on the neurodevelopment of preschool-aged children in Kinshasa, Democratic Republic of the Congo. *Pediatrics* 2008; **122**(1): e123-8.
24. Van Rie A, Dow A, Mupuala A, Stewart P. Neurodevelopmental trajectory of HIV-infected children accessing care in Kinshasa, Democratic Republic of Congo. *Journal of acquired immune deficiency syndromes* 2009; **52**(5): 636-42.
25. Wedderburn CJ, Yeung S, Rehman AM, et al. Neurodevelopment of HIV-exposed uninfected children in South Africa: outcomes from an observational birth cohort study. *Lancet Child Adolesc Health* 2019; **3**(11): 803-13.
26. Wu J, Li J, Li Y, et al. Neurodevelopmental outcomes in young children born to HIV-positive mothers in rural Yunnan, China. *Pediatr Int* 2018; **60**(7): 618-25.
27. Alcaide ML, Rodriguez VJ, Abbamonte JM, et al. Maternal Factors Associated With Infant Neurodevelopment in HIV-Exposed Uninfected Infants. *Open forum infectious diseases* 2019; **6**(10): ofz351.
28. Caniglia EC, Patel K, Huo Y, et al. Atazanavir exposure in utero and neurodevelopment in infants: a comparative safety study. *Aids* 2016; **30**(8): 1267-78.
29. Cassidy AR, Williams PL, Leidner J, et al. In Utero Efavirenz Exposure and Neurodevelopmental Outcomes in HIV-exposed Uninfected Children in Botswana. *The Pediatric infectious disease journal* 2019; **38**(8): 828-34.
30. Chaudhury S, Mayondi GK, Williams PL, et al. In-utero exposure to antiretrovirals and neurodevelopment among HIV-exposed-uninfected children in Botswana. *Aids* 2018; **32**(9): 1173-83.
31. Kacanek D, Williams PL, Mayondi G, et al. Pediatric Neurodevelopmental Functioning After In Utero Exposure to Triple-NRTI vs. Dual-NRTI + PI ART in a Randomized Trial, Botswana. *Journal of acquired immune deficiency syndromes* 2018; **79**(3): e93-e100.
32. Rajan R, Seth A, Mukherjee SB, Chandra J. Development assessment of HIV exposed children aged 6-18 months: a cohort study from North India. *AIDS care* 2017; **29**(11): 1404-9.
33. Rice ML, Zeldow B, Siberry GK, et al. Evaluation of risk for late language emergence after in utero antiretroviral drug exposure in HIV-exposed uninfected infants. *The Pediatric infectious disease journal* 2013; **32**(10): e406-13.
34. Rice ML, Russell JS, Frederick T, et al. Risk for Speech and Language Impairments in Preschool Age HIV-exposed Uninfected Children With In Utero Combination Antiretroviral Exposure. *The Pediatric infectious disease journal* 2018; **37**(7): 678-85.
35. Smith ML, Puka K, Sehra R, Read SE, Bitnun A. Longitudinal development of cognitive, visuomotor and adaptive behavior skills in HIV uninfected children, aged 3-5 years of age, exposed pre- and perinatally to anti-retroviral medications. *AIDS care* 2017; **29**(10): 1302-8.
36. Donald KAM, Fernandez A, Claborn K, et al. The developmental effects of HIV and alcohol: a comparison of gestational outcomes among babies from South African communities with high prevalence of HIV and alcohol use. *AIDS research and therapy* 2017; **14**(1): 28.
37. le Roux SM, Abrams EJ, Donald KA, et al. Growth trajectories of breastfed HIV-exposed uninfected and HIV-unexposed children under conditions of universal maternal antiretroviral therapy: a prospective study. *Lancet Child Adolesc Health* 2019; **3**(4): 234-44.
38. Jumare J, Datong P, Osawe S, Okolo F, Inyang B, Abimiku A. Compromised growth among HIV exposed compared to unexposed children in Nigeria. *Journal of acquired immune deficiency syndromes* 2019; **81**(Supplement 1): 68.
39. Filteau S, Baisley K, Chisenga M, Kasonka L, Gibson RS, Team CS. Provision of micronutrient-fortified food from 6 months of age does not permit HIV-exposed uninfected Zambian children to catch up in growth to HIV-unexposed children: a randomized controlled trial. *Journal of acquired immune deficiency syndromes* 2011; **56**(2): 166-75.
40. Neri D, Somarriba GA, Schaefer NN, et al. Growth and body composition of uninfected children exposed to human immunodeficiency virus: comparison with a contemporary cohort and United States National Standards. *The Journal of pediatrics* 2013; **163**(1): 249-54 e1-2.
41. Aizire J, Sikorskii A, Ogwang LW, et al. Decreased growth among antiretroviral drug and HIV-exposed uninfected versus unexposed children in Malawi and Uganda. *Aids* 2020; **34**(2): 215-25.
42. Tran LT, Roos A, Fouche JP, et al. White Matter Microstructural Integrity and Neurobehavioral Outcome of HIV-Exposed Uninfected Neonates. *Medicine (Baltimore)* 2016; **95**(4): e2577.
43. Spaulding AB, Yu Q, Civitello L, et al. Neurologic Outcomes in HIV-Exposed/Uninfected Infants Exposed to Antiretroviral Drugs During Pregnancy in Latin America and the Caribbean. *AIDS research and human retroviruses* 2016; **32**(4): 349-56.
44. Pintye J, Langat A, Singa B, et al. Maternal Tenofovir Disoproxil Fumarate Use in Pregnancy and Growth Outcomes among HIV-Exposed Uninfected Infants in Kenya. *Infect Dis Obstet Gynecol* 2015; **2015**(276851): 276851.
45. Siberry GK, Williams PL, Mendez H, et al. Safety of tenofovir use during pregnancy: early growth outcomes in HIV-exposed uninfected infants. *Aids* 2012; **26**(9): 1151-9.

46. Jacobson DL, Kunjal P, Williams PL, et al. Growth at 2 years of age in HIV-exposed uninfected children in the United States by trimester of maternal antiretroviral initiation. *Pediatric Infectious Disease Journal* 2017; **36**(2): 189-97.
47. Williams PL, Yildirim C, Chadwick EG, et al. Association of maternal antiretroviral use with microcephaly in children who are HIV-exposed but uninfected (SMARTT): a prospective cohort study. *The Lancet HIV* 2020; **7**(1): e49-e58.
